# Dl-3-n-Butylphthalide Reduces Cognitive Impairment Induced by Chronic Cerebral Hypoperfusion Through GDNF/GFRα1/Ret Signaling Preventing Hippocampal Neuron Apoptosis

**DOI:** 10.3389/fncel.2019.00351

**Published:** 2019-08-13

**Authors:** Wenxian Li, Di Wei, Jiaxing Lin, Jianye Liang, Xiaomei Xie, Kangping Song, Li’an Huang

**Affiliations:** ^1^Department of Neurology, The First Affiliated Hospital, Jinan University, Guangzhou, China; ^2^Department of Neurology, The Second Affiliated Hospital, Xi’an Jiaotong University, Xi’an, China; ^3^Department of Urology, Xijing Hospital, Fourth Military Medical University, Xi’an, China; ^4^Medical Imaging Center, The First Affiliated Hospital, Jinan University, Guangzhou, China

**Keywords:** chronic cerebral hypoperfusion, hippocampus neuron apoptosis, vascular dementia, Dl-3-n-butylphthalide, GDNF/GFRα1/Ret, antibody microarrays

## Abstract

Hippocampal neuron death is a key factor in vascular dementia (VD) induced by chronic cerebral hypoperfusion (CCH). Dl-3-n-butylphthalide (NBP) is a multiple-effects drug. Therefore, the potential molecular mechanisms underlying CCH and its feasible treatment should be investigated. This study had two main purposes: first, to identify a potential biomarker in a rat model of CCH induced VD using antibody microarrays; and second, to explore the neuroprotective role of NBP at targeting the potential biomarker. Glial cell line-derived neurotrophic factor (GDNF)/GDNF family receptor alpha-1 (GFRα1)/receptor tyrosine kinase (Ret) signaling is altered in the hippocampus of CCH rats; however, NBP treatment improved cognitive function, protected against hippocampal neuron apoptosis *via* regulation of GDNF/GFRα1/Ret, and activated the phosphorylation AKT (p-AKT) and ERK1/2 (p-ERK1/2) signaling. We also found that 1 h oxygen-glucose deprivation (OGD) followed by 48 h reperfusion (R) in cultured hippocampal neurons led to downregulation of GDNF/GFRα1/Ret. NBP upregulated the signaling and increased neuronal survival. Ret inhibitor (NVP-AST487) inhibits Ret and downstream effectors, including p-AKT and p-ERK1/2. Additionally, both GDNF and GFRα1 expression are markedly inhibited in hippocampal neurons by coincubation with NVP-AST487, particularly under conditions of OGD/R. GDNF/GFRα1/Ret signaling and neuronal viability can be maintained by NBP, which activates p-AKT and p-ERK1/2, increases expression of Bcl-2, and decreases expression of Bax and cleaved caspase-3. The current study showed that GDNF/GFRα1/Ret signaling plays an essential role in the CCH induced VD. NBP was protective against hippocampal neuron apoptosis, and this was associated with regulation of GDNF/GFRα1/Ret and AKT/ERK1/2 signaling pathways, thus reducing cognitive impairment.

## Introduction

Vascular dementia is widely recognized as the second most common type of dementia. VD is primarily related to diverse cerebrovascular diseases, such as CCH (Du et al., 2017; Duncombe et al., 2017; Yin et al., 2018). CCH is induced by chronic, moderate and persistent deficit of CBF, increasing evidence from laboratorial and clinical researches has indicated CCH is a robustly common factor in pathogenesis of cerebrovascular diseases and neurodegenerative disorders, results in the development and progression of cognitive impairment, such as VD (Du et al., 2017). But to date, it has been difficult to modulate the complex pathological changes caused by CCH (Zhang N. et al., 2017). Model animals of BCCAO are often used to study VD resulting from hypoperfusion (Du et al., 2017). The vascular hypothesis suggests that moderate and persistent cerebral hypoperfusion leads to vasculotoxic and neurotoxic effects due to diminished CBF and prolonged hypoxia, which promotes neurodegeneration and cognitive impairment (Raz et al., 2016). In the neurovascular units of the brain, neurons are the electrically functional cells, and demand a continuous supply of glucose and oxygen (Mao et al., 2019). Neuronal death, a main cause of neuronal loss, is a key hallmark in CCH-related VD (Chen et al., 2017). The hippocampus has critical roles in cognitive processes of learning, memory consolidation, and information retrieval (Wittenberg et al., 2002). Therefore, there may be a correlation between hippocampal neuron vulnerability to CCH induced cognitive damage. The restoration of CBF and rescue of dying neurons represent two primary therapeutic measures. There is uncertainty regarding the exact time of clinical occurrence in hypoperfusion, the irreversibility of neuron damage after reperfusion; therefore, understanding the molecular mechanisms underlying hippocampal neuron death in CCH would be more invaluable for the development of new therapeutic approaches to alleviate vascular cognitive impairment.

Dl-3-n-butylphthalide is a synthetic chiral compound based on l-3-n-butylphthalide (Supplementary Figure S1a), which was originally isolated from the seeds of *Apium graveolens* (Wang et al., 2018). NBP is a multi-target drug in many neurological diseases. For example, NBP has been approved in China to treat ischemic stroke (Qin et al., 2018; Wang et al., 2018). Further, it shows neuroprotective effects in AD (Wang et al., 2016), PD (Xiong et al., 2012), neurotoxicity (Zhao et al., 2016), traumatic brain injury (Zhao et al., 2017), and spinal cord injury (He et al., 2017). NBP is protective against cognitive impairment induced by CCH by increasing sonic hedgehog/patched 1 pathway markers and decreasing endoplasmic reticulum stress-related markers (Niu et al., 2018), or activating the AKT/Nrf2 pathway in the hippocampus of rats (Qi et al., 2018). Our previous work has revealed that NBP can quickly accelerate a recovery in CBF and improvements in cognitive function (Xiong et al., 2017; Li et al., 2019). In addition, in clinical individuals with subcortical vascular cognitive impairment without dementia, NBP improves cognitive function with good safety (Jia et al., 2016). However, potential therapeutic targets of NBP against hippocampal neuron apoptosis have not been fully elucidated in CCH induced cognitive impairment. A greater understanding of NBP would enable a safe combination of NBP with other strategies to treat cerebrovascular diseases.

The quest for suitable drug candidates can be daunting when using traditional screening methods. Identifying a few biologically active compounds from huge libraries of biomolecules is similar to searching for a needle in a haystack. High-efficiency protein microarray technology can identify disease- or drug-related biomarkers more effectively than these traditional methods (Sun et al., 2013). In this study, using an antibody-microarray method, we propose a proteomic strategy that can robustly identify DEPs associated with NBP treatment in a CCH rat model of VD. The functions of the DEPs were further assessed by constructing a PPI network and by biological function enrichment analysis. In addition, the potential neuroprotective targets of NBP will be explored. We aimed to obtain better insight into the mechanisms-of-action of NBP and clarify the potential therapeutic target in improving cognitive function in CCH.

## Materials and Methods

**FIGURE 1:**
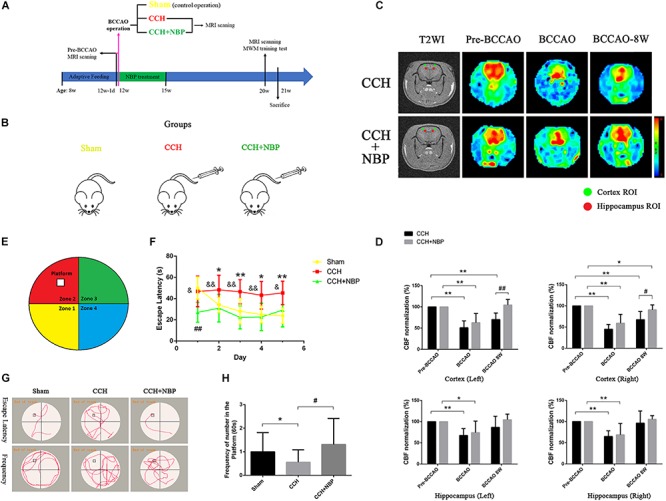
**(A)** The experimental flowchart *in vivo*. Eight-week-old adult male SD rats were obtained and kept for 4 weeks of adaptive feeding. The day before 12 weeks of age (12w-1d), MRI scanning was performed, and the CBF was obtained before the operation (pre-BCCAO). At 12 weeks of age, the operation was performed and the CBF was detected using MRI (15 min after operation). Rats were randomly divided into three groups: Sham (control operation), the bilateral carotid arteries were separated as in the BCCAO group, without being ligated; CCH (BCCAO operation), the bilateral carotid arteries were double-ligated; CCH + NBP: BCCAO operation combined with consecutive NBP treatment for 3 weeks. Eight weeks after the BCCAO operation (20 weeks old), behavioral function was evaluated in the MWM test, and CBF was detected using MRI scanning. Rats were sacrificed for further experiments (21 weeks old). **(B)** Animal groupings used in this study. Rats were randomly divided into three groups: Sham, without ligated; CCH, 8 weeks after double-ligated operation, injection with solvent; CCH + NBP: NBP injection for early 3 weeks with 8 weeks after BCCAO operation. **(C)** Changes in CBF in the cortex and hippocampus were showed by MRI images. Color images are from 3D ASL, gray images from T2WI. Red and green circles indicate detected ROIs of CBF in the hippocampus and cortex, respectively. Histogram showing quantitative results in the different groups after BCCAO operation. **(D)** CBF in the bilateral cortex and hippocampus decreased immediately following BCCAO and 8 weeks after BCCAO, compared to pre-occlusion CBF (*P* < 0.01). CBF increased in the NBP treatment group compared to the BCCAO group. (^*^P < 0.05, ^∗∗^*P* < 0.01, compared to the pre-BCCAO; ^#^*P* < 0.05, ^##^*P* < 0.01, the NBP treatment compared to the CCH). **(E)** Diagram of the MWM. The four colors divide the round swimming pool (the large circle) into four quadrants (Yellow, 1st quadrant; Red, 2nd quadrant; Green, third quadrant; Blue, fourth quadrant). The empty square represents the platform in the 2nd quadrant. **(F)** Escape latency changes in the different groups from day 1 to day 5 (^*^P < 0.05, ^∗∗^*P* < 0.01, CCH group vs. sham group; ^##^*P* < 0.01, CCH + NBP group vs. sham group; ^&^*P* < 0.05, ^&&^*P* < 0.01, CCH vs. CCH + NBP group). **(G)** Swimming path of rats at day 6. The swimming path distance in the platform quadrant was reduced in the CCH. NBP treatment increased the frequency at the platform. **(H)** Quantified changes in the frequency at the platform between the three groups (^*^P < 0.05, CCH group vs. the sham group; ^#^*P* < 0.05, CCH vs. CCH + NBP group). CCH: CCH 8w; CCH + NBP: CCH 8w + NBP.

Sprague Dawley (SD) rats (200–250 g), adult male, aged 8 weeks, were housed at 25 ± 2°C and 40–70% humidity with a cycle of 12-h light/dark for 4 weeks of adaptive feeding. Food and water were provided *ad libitum*. All animal protocols were approved by the institutional animal care committee of the experimental animal management center of Jinan University (Guangzhou, China), and conformed to internationally ethical standards (Guide for the Care and Use of Laboratory Animals. United States NIH Publication 86-23, revised 1985). A total of 97 rats were used. A complete flow chart for this study and the experimental groupings used are shown in Figures 1A,B.

### Animal Surgical Procedures and Drug Administration

Bilateral common carotid artery occlusion operation has been proposed to reproduce the effects of CCH rat model (Zhang et al., 2017; Mao et al., 2019). Each rat was anesthetized with 3% sodium pentobarbital (0.2 ml/kg, intraperitoneal injection, Sigma-Aldrich, United States). A minimally invasive median incision was made in the neck carefully. The bilateral common carotid arteries and the vagus nerves were separated and isolated. For the BCCAO group (total *n* = 70; NBP-treated group, *n* = 28; CCH-treated group, *n* = 42), the bilateral carotid arteries were double-ligated with 2-0 sutures. For the sham group (Sham, *n* = 27), the bilateral carotid arteries were separated, without being ligated. The body temperature of the rats was maintained during surgery, as well as during recovery from anesthesia.

Dl-3-n-butylphthalide-treated rats received daily tail-vein injections (NBP solution: NBP in 2-hydroxypropyl-β-cyclodextrin (HP-β-CD) and 0.9% saline; CSPC^®^ NBP pharmaceutical Co. Ltd., Shijiazhuan, China) for 21 days (3 weeks) [from day 1 after the surgery to day 21, 5 mg/kg/day; for medication dose refer to our previous research (Xiong et al., 2017)]. NBP was injected 1 h before the operation.

### Magnetic Resonance Imaging Measurements

Magnetic resonance imaging were conducted using a Discovery 750 3.0-T scanner (GE Healthcare, United States). NBP-treated (*n* = 8) and CCH-treated (*n* = 8) rats were scanned.

Following anesthesia, animals were placed in a prone position before scanning. All imaging parameters for the 3D ASL series, and CBF calculation methods were automatically recorded, as described in previous studies (Xiong et al., 2017). The regions of interests (ROIs) (area: 4 mm^2^) were measured in the bilateral hippocampus and cortex; three values were selected on each side to calculate an average value.

### Morris Water Maze Test

Rat behavioral function was evaluated in the MWM test (Figure 1E). A total of 44 rats were assessed [Sham, *n* = 7; 2 weeks (w) after BCCAO (CCH 2w), *n* = 8; CCH 4w, *n* = 9; CCH 8w, *n* = 8; CCH 8w + NBP (CCH + NBP) group, *n* = 12].

A blind test was performed prior to the experimental task to exclude blind rats. The utilized maze was a round tank, divided into four quadrants, 150 cm in diameter and 100 cm deep, filled up to a depth of 40 cm with tepid water (25 ± 1°C). A movable square platform, 10 cm in diameter, was located 5 cm below the water surface. The maze was surrounded by white paper, on which black visual stimuli of four different shapes and sizes were placed in the four quadrants. Every trial started at a different point, alternating between the four quadrants. A camera was located above the center of the maze that relayed images to a videocassette recorder and the Image Analysis Computer System (Ethovison XT, Noldus Information Technology Co., Hague, Netherlands). Each test consisted of four trials from the four quadrants per day, and was conducted on five consecutive days. The hidden platform was always located in the 2nd quadrant of the water maze. Rats were submerged gently into the water, facing toward the inside wall of the tank. In the maze, rats were allowed to swim for a maximum time of 60 s. Rats were allowed to remain on the platform for 20 s at the end of each trial. Performance was evaluated for EL time for all trials. The platform was withdrawn at the sixth day of training. Swimming time spent (60 s) in the target quadrant with the retracted platform, and the frequency of time in the target quadrant, were used as parameters for retention of spatial memory.

### Antibody-Microarray Technology

Fresh frozen hippocampal tissue was assayed using antibody-based protein microarrays [Sham group: *n* = 2 (four samples merged into two samples); CCH 8w (CCH) group: *n* = 3; CCH 8w + NBP (CCH + NBP) group: *n* = 3]. We used a commercially available microarray (AAR-BLG-1, Biotin label-based rat antibody array, RayBiotech^®^, United States) containing 90 antibodies, including 16 classifications, such as neurokines, chemokines, and cytokines, as well as another 12 control antibodies. Manufacturer’s protocol was described previously (Li et al., 2019).

We prepared a protease inhibitor cocktail and a cell lysis buffer at a ratio of 1:99, which was added to the hippocampal tissue on the ice. After 30 min, samples were spun down in a refrigerated centrifuge, at 13000 rpm, for 20 min. We then extracted the supernatant. The minimum protein concentration of the samples was 15000 μg/ml, as determined by comparison to a bovine serum antigen (BSA) standard. Next, all the samples were taken at 2 mg/ml, 50 μL, in the dialysis tubes. The tubes were then placed in 1 × phosphate buffered saline (PBS; pH = 8, 4000 ml) at 4°C, with stirring, and the dialysis fluid was changed at intervals of 3 h.

During biotin labeling, reagent contamination was prevented by addition of amines or sodium azide (e.g., Tris, glycine). After the glass assay chips were left at 20–25°C for 20–30 min, we removed the sealing strips and placed the chips in a vacuum dryer or at room temperature for 1–2 h. In each well on the chip, 400 μL of 1 × sealing solution was added and incubated at room temperature for 1 h to avoid bubbles. Then, we removed the fluid and added 400 μL of sample to each well, with one array per sample, and incubated the arrays at 4°C, overnight, with constant shaking. Then, the samples were removed, and 1 × washing solution was added to each well and incubated at room temperature with constant shaking. The chips were washed four times for 5 min each. After repeated washing, the washing solution was removed, and 400 μL of a diluted fluorescent agent, streptavidin with Cy3, was added to each well. The arrays were covered with an aluminum foil seal, and samples were incubated, in the dark, at room temperature, for 2 h. After incubation, the arrays were again washed with washing solution.

Using a laser scanner (InnoScan 300 Microarray Scanner; Innopsys; Parc d’Activités Activestre; 31 390 Carbonne, France), fluorescence was measured in the Cy3, or green, channel (excitation frequency = 532 nm; resolution: 10 μm). Data were extracted by the original analysis software of the instrument. To determine spot intensities, the mean pixel intensity per spot was calculated. To determine background intensities, the median pixel intensity per background “doughnut” was calculated. Individual array spots were background subtracted locally (by subtracting the median background across spot replicates in each sample).

### Protein–Protein Interaction Network Construction by STRING

Through inputting the ID number of DEP, DEPs were analyzed by the online tool, STRING (a database of known and predicted protein interactions)^1^, to predict interactions. A combined score of >0.9 (a high confidence score) was considered significant.

### Functional Analysis of DEPs

Conservation analysis of the DEPs in rats and humans was conducted using BLAST^®2^ and NCBI-Gene^3^.

Biological functional enrichment analyses of the DEPs, including GO functional analysis and KEGG pathway analysis, were conducted. In the GO analysis, the categories used included CC, BP, and MF terms. *P* < 0.05 was considered a statistically significant difference. In the KEGG pathways analysis, enriched pathways were identified according to the hypergeometric distribution with *P* < 0.05.

### Real-Time Quantitative PCR

For each sample, cDNA (2 μL) was quantified and duplicated using Light Cycler 480 SYBR Green I on a Light Cycler 480 II as per manufacturer instructions. Cycling conditions: 10 min at 95°C, 40 cycles of 15 s at 95°C, 60 s at 60°C. Melt curve cycles were immediately performed and the cycling conditions were as follows: 5 s at 95°C, 60 s at 60°C, then a gradual temperature rise to 95°C at a rate of 0.3°C/s, followed by 15 s at 60°C. Melt curve analysis was performed to verify primer specificity. Data are displayed as a fold change above the proliferative condition mRNA levels using 2^∧(ΔΔCt)^ values.

Primer sequences:

Glial cell line-derived neurotrophic factor (GDNF):

R-GDNF-S: TGTCTGCCTGGTGTTGCTCC

R-GDNF-A: TCTTCGGGCATATTGGAGTCAC

GDNF family receptor alpha 1 (GFRα1)

R-GFRα1-S: CCAACTACGTAGACTCCAGCAGC

R-GFRα1-A: GGTCACATCTGAGCCATTGCC

Receptor tyrosine kinase (Ret)

R-Ret-S: CGTACATCGAGACTTAGCTGCC

R-Ret-A: TCCCATAGCAGCACTCCAAAG

Neural cell adhesion molecule (NCAM)

R-NCAM-S: CGCCGAGTACGAAGTATATGTGG

R-NCAM-A: TGACCACCAGGAGTAGGACGAA

GAPDH

R-GAPDH-S: TTCCTACCCCCAATGTATCCG

R-GAPDH-A: CATGAGGTCCACCACCCTGTT

### Western Blot

Fresh hippocampal tissue samples were a random subset picked blindly. All tissues were lysed in RIPA buffer cocktail (containing protease and phosphatase inhibitors) (Cell Signaling Technology, United States). Total protein concentrations were determined with a BCA Protein Assay Kit (Thermo Fisher Scientific, United States). Total protein (10–20 μg) was loaded for each sample into pre-cast 4–12% bis-tris gels and run with MOPS buffer (Invitrogen, United States). The proteins were transferred onto polyvinylidene fluoride membranes (Millipore, United States). The membranes were incubated overnight at 4°C with antigen-specific primary antibodies and detected with species-specific horseradish-peroxidase-labeled secondary antibodies. An ECL western blotting detection kit (GE Healthcare) was used to obtain a chemiluminescence signal, which was detected using Amersham Hyperfilm ECL (GE Healthcare). Bands of interest were normalized to β-actin (1:3000, Abcam, Cambridge, MA, United States, ab8226) for a loading control. For GDNF we used anti-GDNF antibodies (1:1000, Abcam, Cambridge, MA, United States, ab18956); GFRα 1 (1:1000, Abcam, Cambridge, MA, United States, ab186855); Ret (1:1000, Abcam, Cambridge, MA, United States, ab134100); NCAM (1:1000, Abcam, Cambridge, MA, United States, ab9018); Bcl-2 (1:1000, Abcam, Cambridge, MA, United States, ab59348); Bax (1:1000, Abcam, Cambridge, MA, United States, ab182733); Caspase 3 (1:500, Cell Signaling Technology, United States, #9662); Cleaved caspase 3 (1:1000, Cell Signaling Technology, United States, #9664); t-AKT (1:1000, Cell Signaling Technology, United States, #9272); p-AKT [phospho-AKT(Ser473), 1:1000, Cell Signaling Technology, United States, #9271]; t-ERK1/2 [ERK1(pT202/pY204) + ERK2(pT185/pY187), 1:2000, Abcam, Cambridge, MA, United States, ab184699]; p-ERK1/2 [Anti-ERK1(pT202/pY204) + ERK2(pT185/pY187), 1:2000, Abcam, Cambridge, MA, United States, ab76299]; HIF-1α (1:1000, Abcam, Cambridge, MA, United States, ab1).

These experiments were performed in triplicate, and the protein bands were quantitatively analyzed with AlphaEase FC software.

### Immunostaining

Seven days after the behavioral experiments, rats were perfused transcardially with a 0.9% physiological saline solution and, subsequently, put under deep anesthesia with 4% paraformaldehyde. After the process of perfusion, the brains were removed and immersed in 4% paraformaldehyde immediately for 48 h, then embedded in paraffin. To ensure matching of hippocampal sections between groups, we used anatomical landmarks provided by the brain atlas.

Coronal brain sections (selected areas, Supplementary Figure S1b), 5 μm in thickness, were stained with hematoxylin-eosin (HE) and Nissl’s staining. Sections were labeled with CD34 (for endothelial cells of microvessels), NeuN (for neurons), glial fibrillary acidic protein (GFAP) (for astrocytes), Iba1 (for microglial cell), and 4′,6-diamidino-2-phenylindole dihydrochloride (DAPI) (for the cell nucleus) antibodies. First, sections were immersed in a blocking solution at room temperature for 2 h, and then incubated overnight at 4°C with either CD34 antibody (rabbit, 1:200, Abcam, Cambridge, MA, United States, ab81289), NeuN antibody (mouse, 1:200, Abcam, Cambridge, MA, United States, ab104224), GFAP antibody (mouse, 1:200, Abcam, Cambridge, MA, United States, ab10062), or Iba1 antibody (mouse, 1:200, Abcam, Cambridge, MA, United States, ab15690). Sections labeled with NeuN/GFAP/Iba1 antibodies, and GDNF (rabbit, 1:200, Abcam, Cambridge, MA, United States)/GFRα 1 antibodies (rabbit, 1:200, Abcam, Cambridge, MA, United States)/Ret (rabbit, 1:200, Abcam, Cambridge, MA, United States) were counterstained with DAPI. After three washes with PBS, sections were then incubated with the appropriate secondary antibody (1:500) for 2 h at room temperature. After three washes in PBS, sections were mounted with a fluorescence antifade mounting medium. For each type of labeling, *n* = 3 rats from each of the sham, BCCAO 8w, and NBP groups were used. Digital images were captured from the CA1 region at 40 × 10 magnification, and from the CA3 region and dentate gyrus (DG) at 20 × 10 magnification. Three images were captured from both sides of each region in each group, separately. The number of positive cells was counted with Image J and Image-Pro Plus 6.0 For each photograph, the exposure time was consistent. Two experienced pathology experts, blinded to all experimental information, independently measured and interpreted the final results.

For cell immunofluorescence assay, primary neurons were fixed in 4% paraformaldehyde for 20 min at room temperature. After incubation in 1% BSA, 5% goat serum, and 0.2% Triton X-100 in PBS for 2 h at 4°C. GDNF (rabbit, 1:200, Abcam), GFRα1 (rabbit, 1:200, Abcam), Ret (rabbit, 1:200, Abcam), and NeuN (mouse, 1:200, Abcam) antibodies were sequentially applied overnight at 4°C. Corresponding secondary antibodies were applied for 2 h at room temperature, followed by counterstaining with DAPI. For different neuron specimens (*n* = 3 tests per group), digital images were captured at 40 × 10 magnification. The number of neurons in each image were counted with Image J and Image-Pro Plus 6.0.

### TUNEL Staining

The TdT-mediated dUTP Nick-End Labeling (TUNEL) assay was performed according to the manufacturer’s protocol (DeadEnd^TM^ Fluorometric TUNEL System, Promega, Madison, WI, United States). The cerebral sections or cell specimens were incubated with a proteinase K solution for 15 min at 37°C to enhance permeability. The sections were then incubated with the TUNEL reaction mixture for 1 h at 37°C. After being rinsed with PBS, the sections were mounted using an antifade mounting media containing DAPI. Images were acquired using a fluorescent microscope (Nikon, Japan), the number of positive TUNEL cells were counted with Image J and Image-Pro Plus 6.0.

### *In situ* Hybridization

To detect the expression and location of GFRα1 and GDNF, three rats were randomly chosen from the NBP group for *in situ* hybridization and immunohistochemistry. The hippocampus of the rats were harvested and cut into 20-μm thick sections. The sections were fixed in 4% paraformaldehyde and acetylated with 0.25% acetic anhydride. Prior to hybridization with the probes for 12–16 h (37°C), the sections were prehybridized in a hybridization solution without probes. Subsequently, the sections were blocked at 37°C for 1 h, immersed in a second antibody (1: 1000) at 4°C overnight, and finally visualized with a fluorescent microscope (Nikon, Japan).

GDNF probe:

5′-FAM-CCTCTGGCCTCTGCGACCTTTCCCT-FAM-3′

GFRα1 probe:

5′-FAM-GTGCTTGGCCGGAACCTTGTCGA-FAM-3′

### Transmission Electron Microscopy

Fresh hippocampal tissues (*n* = 5 per group) were cut into pieces of 1 mm^3^, and fixed in a 2.5% glutaraldehyde solution overnight at 4°C. Specimens were rinsed with PBS, then soaked in osmium tetroxide. After dehydration in acetone, specimens were embedded in epoxide resin, and 70-nm in thickness for sections. Specimens were then stained with uranyl acetate followed by lead citrate. Lastly, ultrastructural changes of neurons were imaged using a TEM (HT7700, Hitachi, Japan).

### Culture of Primary Hippocampal Neurons

Cultures of primary hippocampal neurons were extracted from E18-20 SD rat embryos as described previously, with some adjustments and improvements (Zhang et al., 2017). In brief, the hippocampus of the embryos (*n* > 20/every time) were dissected in a Dulbecco’s modified Eagle medium (DMEM) (high glucose) solution supplemented with a 10% fetal bovine serum (FBS) (Thermo Fisher Scientific, Gibco, United States) and 1% penicillin-streptomycin (Biological Industries, Israel), at 0–4°C. It was then digested with 0.125% trypsin-EDTA (Thermo Fisher Scientific, Gibco, United States) and 0.4 mg/ml deoxyribonuclease I (Worthington Biochemical Corporation, United States) for 15 min, at 37°C. After centrifugation at 1000 rpm for 5 min, samples were resuspended in a DMEM/F12 medium (Thermo Fisher Scientific, Gibco, United States) supplemented with 10% FBS and 1% penicillin-streptomycin. Viable cells were counted using 0.4% trypan blue (G-clone, Beijing, China) in a hemocytometer (QIUJING, Shanghai, China) and plated at a density of 2 × 10^5^/mL into a 96-well plate pre-coated with poly-L-lysine (Sigma-Aldrich) (8–12 h). After 4 h, the culture medium was replaced with a serum-free neurobasal medium (Thermo Fisher Scientific, Gibco, United States) supplemented with 2% B-27 (Thermo Fisher Scientific, Gibco, United States) and 1% penicillin-streptomycin (Thermo Fisher Scientific, Gibco, United States). Half of the culture medium was replaced with fresh medium every 3 days.

### Oxygen-Glucose Deprivation/Reperfusion and Drug Treatment *in vitro*

Existing studies have adopted OGD/R to simulate CCH model *in vitro* (Zhang et al., 2017; Mao et al., 2019). On day 7, the medium was replaced with DMEM without glucose (Thermo Fisher Scientific, Gibco, United States) and the neurons were incubated in an AnaeroPack^®^-Anaero (MGC AnaeroPack Series, Japan) to induce OGD injury at 37°C for 1 h. The medium was then replaced with serum-free neurobasal medium supplemented with 2% B-27 to induce reperfusion (R). For concurrent treatment, NBP (60 μM) (Supplementary Figures S4f,g) was present in the culture medium during initial OGD 1 h to R 24 h (25 h in total). Then the medium was replaced with serum-free neurobasal medium supplemented with 2% B27. At R 36 h after OGD (at OGD 1 h/R 36 h), Ret inhibitor (Ret i, NVP-AST487, 5 μM, MedChemExpress, MCE, China) was added and cultured for another 12 h (R 36 h to R 48 h) to inhibit expression of Ret (Supplementary Figures S4h,i). NVP-AST487 is a Ret kinase inhibitor, inhibiting Ret autophosphorylation and activation of downstream effectors. In addition, expression of both GDNF and GFRα1 are markedly inhibited by coincubation with NVP-AST487.

### Cell Survival Assays

Neuron viability was assessed by Cell Counting Kit-8 (CCK-8) detection (450 nm) at 48 h after OGD, and before measurement cells were incubated with 10 μL CCK-8 (Dojindo, Japan) in 90 μL fresh serum-free neurobasal complete medium for 4 h at 37°C. All samples were assayed in five replicates and each experiment was repeated at least five times.

### Statistical Analyses

The raw data obtained from the scanning of the antibody microarray were acquired by Raybiotech^®^ software and normalized between the microarrays. For cell quantification (TUNEL and immunofluorescence), three rats in each group were used *in vivo*, three slices per animal were stained, three images were analyzed per slice (CA1/CA3/DG section); *in vitro* model, three to five images per well were taken, and three repetitions in each experiment. Cells were counted (automatically or manually) and statistically analyzed.

All other statistical analyses were performed using SPSS 19.0 (Abacus Concepts Inc., Chicago, IL, United States). Data were represented as the mean ± standard deviation (SD). Statistical analyses for differences between groups were performed using the two independent samples *t* test. Statistical differences among the groups were assessed by one-way ANOVA, when homogeneity of variance was determined, Fisher’s least significant difference (LSD) test was used; when homogeneity of variance was not determined, Tamhane’s T2 test was used. *P* < 0.05 was considered statistically significant.

## Results

### *In vivo* Experiments

#### NBP Promoted Recovery of CBF After BCCAO

In this study, we used the 3D ASL to dynamic observation. CBF was measured in the cortex and hippocampus of rats at six timepoints, including pre-occlusion, BCCAO, and the 1st, 2nd, 4th, and 8th week after BCCAO. In cortical areas, CBF was consistently lower following BCCAO than in the pre-BCCAO state, while CBF was restored in the hippocampus at the BCCAO 8w (Supplementary Figures S1c,d). These results indicated successful establishment of the BCCAO-induced CCH model.

Cerebral blood flow in the hippocampus and cortex of NBP-treated rats was assessed at pre-occlusion, BCCAO operation, and BCCAO 8w, and compared with that in the corresponding CCH-treated rats (Figure 1C). Following BCCAO operation, the CBF decreased immediately after surgery in both the cortex and hippocampal areas (left cortex: CBF decreased to 50.91% ± 15.97 in the CCH-treated and 62.84% ± 21.64 in the NBP-treated groups. Right cortex: CBF decreased to 45.07% ± 11.11 in the CCH-treated and 59.14% ± 20.85 in the NBP-treated groups. Left hippocampus: CBF decreased to 67.52% ± 16.31 in the CCH-treated and 74.13% ± 27.09 in the NBP-treated groups. Right hippocampus: CBF decreased to 64.88% ± 13.32 in the CCH-treated and 68.64% ± 26.91 in the NBP-treated groups). The cortex in the CCH-treated (BCCAO 8w) rats had lower CBF at 8th week after BCCAO, while the cortex in NBP-treated rats showed recovery of CBF [Left cortex: CBF decreased to 70.18% ± 15.25 in the CCH-treated group, compared to pre-BCCAO (*P* < 0.01); CBF was increased to 96.49% ± 10.67 in the NBP-treated group compared BCCAO 8w (*P* < 0.01). Right cortex: CBF decreased to 67.48% ± 19.19 in the CCH-treated compared with pre-BCCAO (*P* < 0.01); CBF was increased to 90.69 ± 11.64% in the NBP-treated group compared to BCCAO 8w (*P* < 0.05)] (Figure 1D). In the hippocampus, CBF was restored to the preoperative level with or without NBP treatment [Left hippocampus: CBF increased to 86.61% ± 26.26 in the CCH-treated and 104.54% ± 13.28 in the NBP-treated groups compared to pre-BCCAO (*P* > 0.05). Right hippocampus: CBF increased to 96.34% ± 28.76 in the CCH-treated and 105.37% ± 8.6 in the NBP-treated groups compared to pre-BCCAO (*P* > 0.05)] (Figure 1D).

These results suggest that NBP treatment promotes recovery of cerebral CBF in CCH rats.

#### A Greater Degree of Cognitive Impairment Is Observed in CCH-Treated Than in NBP-Treated Group

The different experimental groups were tested for learning and memory deficits as well as EL in the MWM test. The frequency of crossing the platform was analyzed.

After the surgery, EL was significantly prolonged at CCH 2w, CCH 4w and CCH 8w (Supplementary Figure S1e). The frequency of crossing the original platform was decreased in the CCH 2w, CCH 4w, and CCH 8w (Supplementary Figure S1f) compared to sham groups.

These above results demonstrated the success establishment of the CCH-induced VD model at BCCAO 8w/CCH 8w.

Escape latency was notably reduced in the NBP-treated group (CCH 8w + NBP) compared to the CCH group (CCH 8w) (day 1, *P* < 0.05; day 2 to day 4, *P* < 0.01; day 5, *P* < 0.05). Rats in the CCH group had significantly increased EL compared to that in the sham rats from day 2 to day 5 (all *P* < 0.05); From day 2 to day 5, EL in NBP-treated rats was comparable to that in sham rats (*P* > 0.05) (Figures 1F,G). In addition, the frequency of crossing the platform at day 6 was significantly decreased in the CCH-treated group compared to both the sham rats and the NBP-treated group (Figures 1G,H).

These above results demonstrated the NBP reduce cognitive impairment in CCH rats model.

#### Angiogenesis in the Hippocampus of Rats With CCH

CD34 immunofluorescence was used to show changes in angiogenesis; we found cortical CD34 positive cells were increased in CCH 8w and NBP-treated groups. However, we did not find any statistical significance in CD34 positive cells between the three groups in the hippocampus (CA1, CA3, and DG areas) (Supplementary Figure S2). Quantitative analysis of data is shown in Supplementary Figures S2a–d.

#### NBP Reduced Hippocampus Neuronal Apoptosis in CCH Model

Hematoxylin-eosin and Nissl’s staining showed neuronal damage in the CA1 and CA3 areas in CCH 4w and CCH 8w rats. This change was not observed in the CCH 2w rats and was most pronounced in the CCH 8w rats (Supplementary Figures S1g,h), suggesting that the process of CCH had led to delayed neuronal death.

**FIGURE 2:**
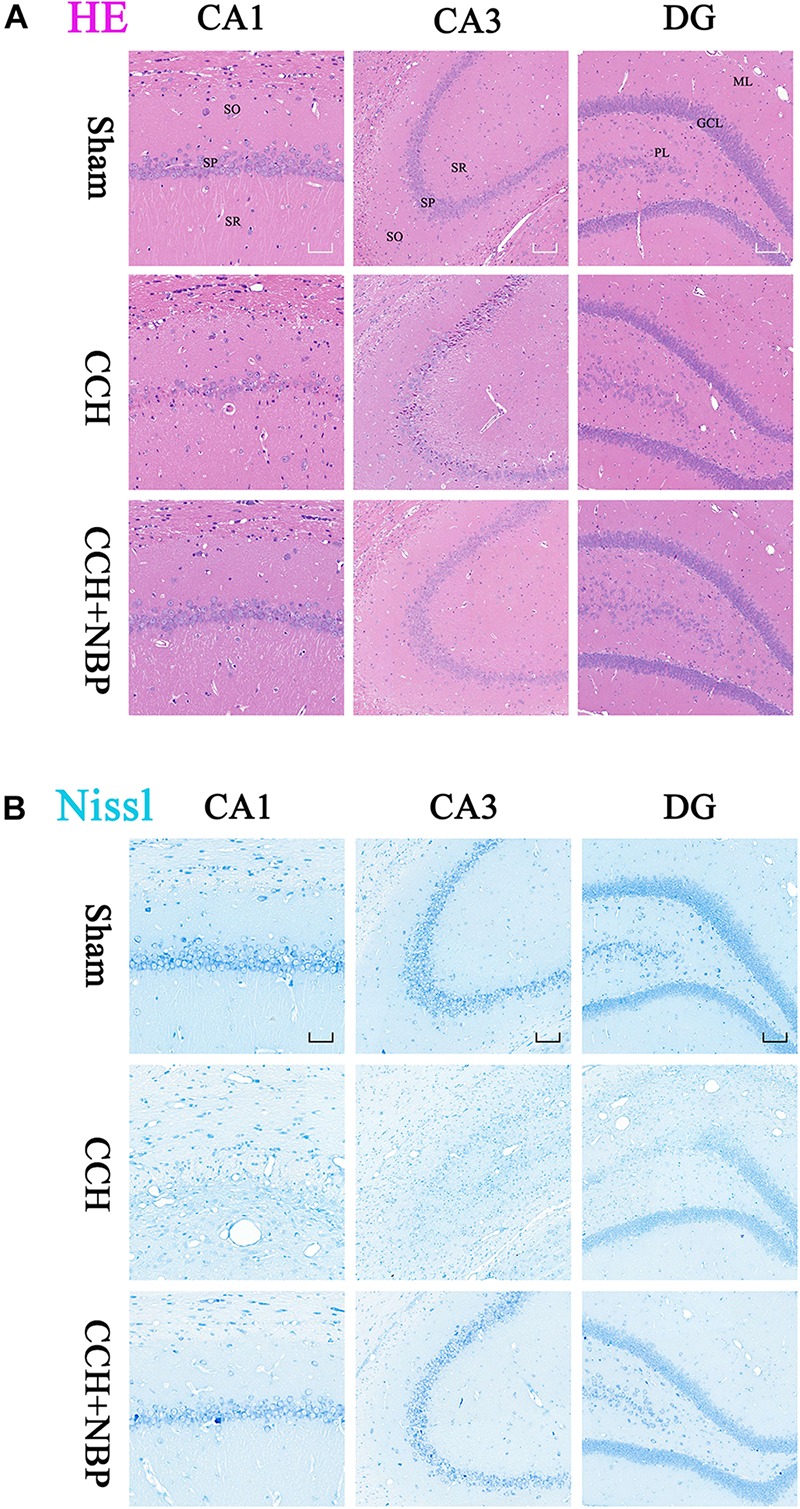
Dl-3-n-butylphthalide improves the pathological changes of the hippocampus. **(A)** Representative photomicrographs of hippocampal HE staining in the three groups. HE staining showed morphological changes in the rat hippocampus. Neuronal damage was detected in the SP in both the CA1 and CA3 areas in the CCH group. NBP treatment significantly increased the number of viable neurons, compared with the CCH group. **(B)** Representative photomicrographs of hippocampal Nissl staining in the three groups. Neuronal damage was detected in both the CA1 and CA3 area, and a partial region of the GL of the DG area in the CCH group. Normal neurons were arranged in an orderly manner, with typical morphology, and an evident nucleus and nucleolus. Abnormal neurons had lost their morphology, shrunken and deeply stained. NBP treatment reduced the damage, both in number of neurons and the morphological structure of the hippocampus. CCH: CCH 8w; CCH + NBP: CCH 8w + NBP. CA, cornus ammonis; DG, dentate gyrus; SO, stratum oriens; SP, stratum pyramidale; SR, stratum radiatum; ML, molecular layer; GL, granule cell layer; PL, polymorphic layer. CA1 area, magnification 200×, scale bar = 50 μm, CA3 and DG areas, magnification 100×, scale bar = 100 μm.

Dl-3-n-butylphthalide treatment improved this outcome, reduced neuronal loss and death (Figures 2A,B).

**FIGURE 3:**
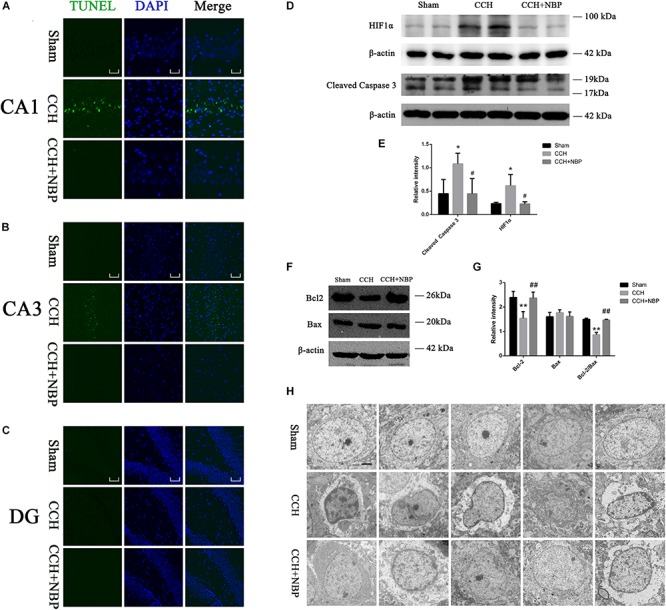
Dl-3-n-butylphthalide improved hippocampal neuronal apoptosis in the CCH model. TUNEL staining (green) shows neuronal apoptosis in the hippocampus (CA1**(A)**, CA3**(B)**, and DG**(C)** areas) of sham, CCH, and CCH + NBP groups at 8 weeks after BCCAO. Nuclei were stained with DAPI (blue). CA1 area, 400× magnification, scale bar = 25 μm. CA3 and DG areas, 200× magnification, scale bar = 50 μm. **(D)** Western blot analysis of the expression of HIF-1α and cleaved caspase-3 *in vivo*. **(E)** The expression of HIF-1α and cleaved caspase-3 were quantified and represented as a histogram. β-actin was used as an internal control. *n* = 3 experiments. ^*^P < 0.05, CCH group vs. sham group; ^#^*P* < 0.05, CCH vs. CCH + NBP group. **(F)** Bcl-2 family proteins are involved in the anti-apoptotic effect of NBP. **(G)** The expression of Bcl-2, Bax and Bcl-2/Bax ratio were quantified and represented as a histogram. β-actin was used as an internal control. *n* = 3 experiments. ^∗∗^*P* < 0.01, CCH group vs. sham group; ^##^*P* < 0.01, CCH vs. CCH + NBP group. **(H)** NBP treatment protects against morphological and ultrastructural changes of neurons in the hippocampus. Sham group: the neurons in the cortex had large round nuclei, the double nuclear membranes were clear and complete. CCH group: neurons were irregular and exhibited chromatin condensation, cytoplasm dissolution, and vacuole formation. The nuclear membranes and organelles of neurons were dissolved or absent. NBP treatment group: the damage to the neurons was alleviated (*n* = 5 per group). Scale bar = 2 μm. CCH: CCH 8w; CCH + NBP: CCH 8w + NBP.

TUNEL staining is a marker for cell apoptosis. TUNEL analysis showed TUNEL-positive cells were widely present in the hippocampus (CA1 and CA3 areas) of the CCH-treated rats, while NBP-treated could significantly reverse the phenomenon (Figures 3A–C).

Western blotting showed that cleaved caspase-3 expression was decreased, however, Bcl-2 expression and Bcl-2/Bax ratio were increased in the sham/NBP-treated group compared to the CCH-treated group (Figures 3D,F). Quantitative analysis of data is shown in Figures 3E,G.

#### Alterations in Hippocampus Neuronal Morphology and Ultrastructure

Normal hippocampal neurons had large oval nuclei with clear nuclear membranes. In the CCH 8w group, neurons had irregular nuclear membranes, chromatin condensation, and many vacuoles. These changes were alleviated in the NBP-treated group (Figure 3H). These results indicate that NBP treatment alleviates hippocampal neuron damage in CCH rats.

#### Networks and Function of DEPs in CCH and NBP Treatment Rats

Identification of DEPs between CCH-treated, NBP-treated, and sham rats was based on the results of protein arrays.

At first, there were 6 DEPs and GFRα1 served as a target protein in CCH 8w and sham groups, because GFRα1 expression was significantly downregulated in the CCH 8w, with a high degree of conservation (Supplementary Figures S3a,b). GO (Supplementary Figures S3c–e) and KEGG (Supplementary Figure S3f) analysis showed these DEPs were involving with some pathways.

**FIGURE 4:**
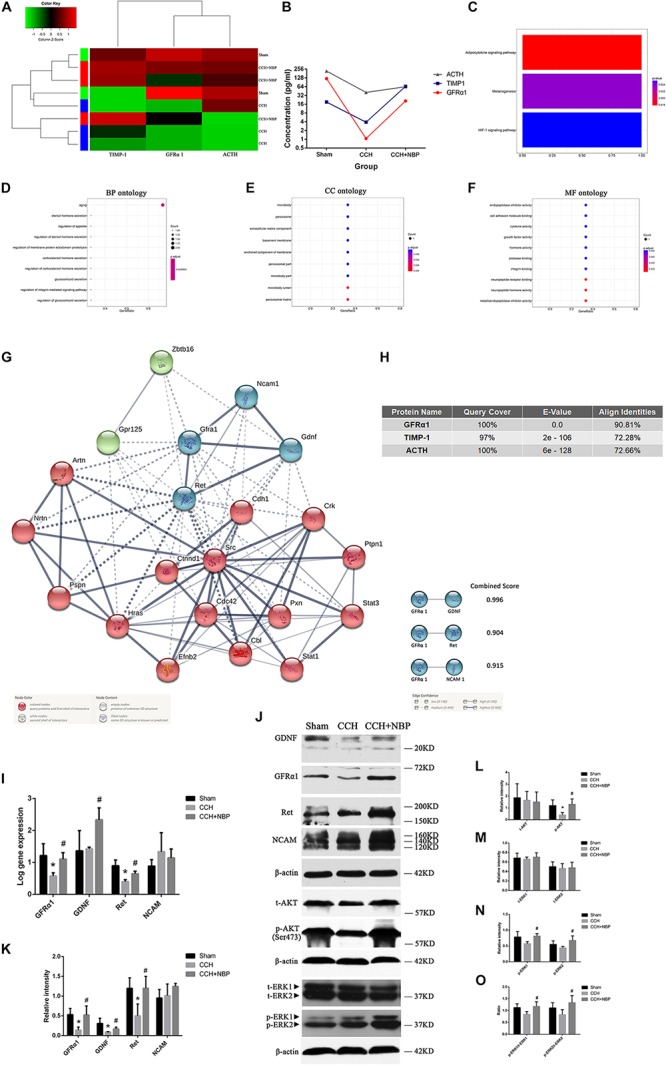
Biological function and validation of DEPs. **(A)** Heat map representation of the protein level analysis showing the top three different proteins (*P* < 0.05), using antibody arrays. **(B)** Concentration of the top three most different proteins in the three groups. **(C)** KEGG pathways of the DEPs. GO enrichment of DEPs: **(D)** BP; **(E)** CC; and **(F)** MF ontology. **(G)** PPI network diagram of the three node proteins, GDNF, Ret, and NCAM, showing more association with GFRα1 (combined score > 0.9), indicating that they have higher hub degrees. **(H)** Conservative analysis of the top three most different proteins (GFRα1, TIMP-1, and ACTH). **(I)** Using (Rt-qPCR, GFRα1/GDNF/Ret/NCAM mRNA expression was detected in the hippocampus of the three groups. *n* = 3 per experiments (^*^P < 0.05, CCH vs. sham group; ^#^*P* < 0.05, CCH vs. CCH + NBP group). **(J)** GDNF/GFRα1/Ret signaling, p-AKT and p-ERK1/2 pathway were significantly downregulated in the CCH group, and upregulated in the sham and NBP treatment groups. **(K)** The relative intensity of GDNF/GFRα1/Ret/NCAM was quantified and represented as a histogram. **(L)** The relative intensities of t-AKT(Ser473) and p-AKT(Ser473) were quantified and represented as a histogram. **(M)** The relative intensities of t-ERK1(pT202/pY204) and t-ERK2(pT185/pY187) were quantified and represented as a histogram. **(N)** p-ERK1(pT202/pY204) and p-ERK2(pT185/pY187) were both decreased in the CCH group, but increased in the NBP treatment group. **(O)** p-ERK1(pT202/pY204)/t-ERK1(pT202/pY204) and p-ERK2(pT185/pY187)/t-ERK2(pT185/pY187) were both decreased in the CCH group, but increased in the NBP treatment group. *n* = 3 per experiment. ^*^P < 0.05, CCH vs. sham group; ^#^*P* < 0.05, CCH vs. CCH + NBP group. CCH: CCH 8w; CCH + NBP: CCH 8w + NBP.)

Then tissues of three groups were compared; the top three DEPs [GFRα1 (*P* < 0.05), TIMP-1 (*P* < 0.05) and ACTH (*P* < 0.05)] were selected for further analysis (Figures 4A,B). The top three pathways revealed by KEGG analysis were the adipocytokine signaling pathway, the melanogenesis signaling pathway, and the HIF-1 signaling pathway (Figure 4C). The GO analysis also showed that the DEPs were associated with the following BP: response to aging, steroid hormone secretion, and regulation of appetite; CC: the microbody lumen and peroxisomal matrix; and MF: neuropeptide hormone activity, neuropeptide receptor binding (Figures 4D–F). Next, using Protein BLAST^®^, the conservation of the three DEPs between *Homo sapiens* and *Rattus norvegicus* was analyzed. The results showed that GFRα1 was the most conserved DEP (Query cover = 100%; *E*-value = 0.0; Align identities score = 90.81%) (Figure 4H).

STRING was used to predict the protein-protein interactions (PPIs) of the DEP. Three node proteins, GDNF, Ret, and NCAM, showed a strong association with GFRα1, indicating that these four hub proteins might play crucial roles in our animal model (Figure 4G). Using the above methodology, we identified highly interconnected clusters of receptors and ligands, GDNF/GFRα1 binding to Ret or NCAM, which could be potential NBP therapy targets in CCH induced VD.

#### NBP-Treatment Activated AKT and ERK1/2 Pathways *in vivo*

AKT and ERK1/2 are two downstream effectors of GDNF/GFRα1/Ret signaling. p-AKT, p-ERK1/2 levels and p-ERK1/2/t-ERK1/2 ratio were decreased in the CCH 8w group but activated in the NBP-treated group (Figure 4J). A quantitative analysis of the data is shown in Figures 4L–O.

#### Hippocampal GDNF/GFRα1/Ret Expression in Different Experimental Groups

Rt-qPCR (Figure 4I) and western blotting (Figure 4J) showed GFRα1, GDNF, and Ret mRNA and protein levels were significantly increased in NBP-treated rats compared to CCH-treated rats. However, NCAM levels were not different between the three groups. A quantitative analysis of western blotting is shown in Figure 4K.

**FIGURE 5:**
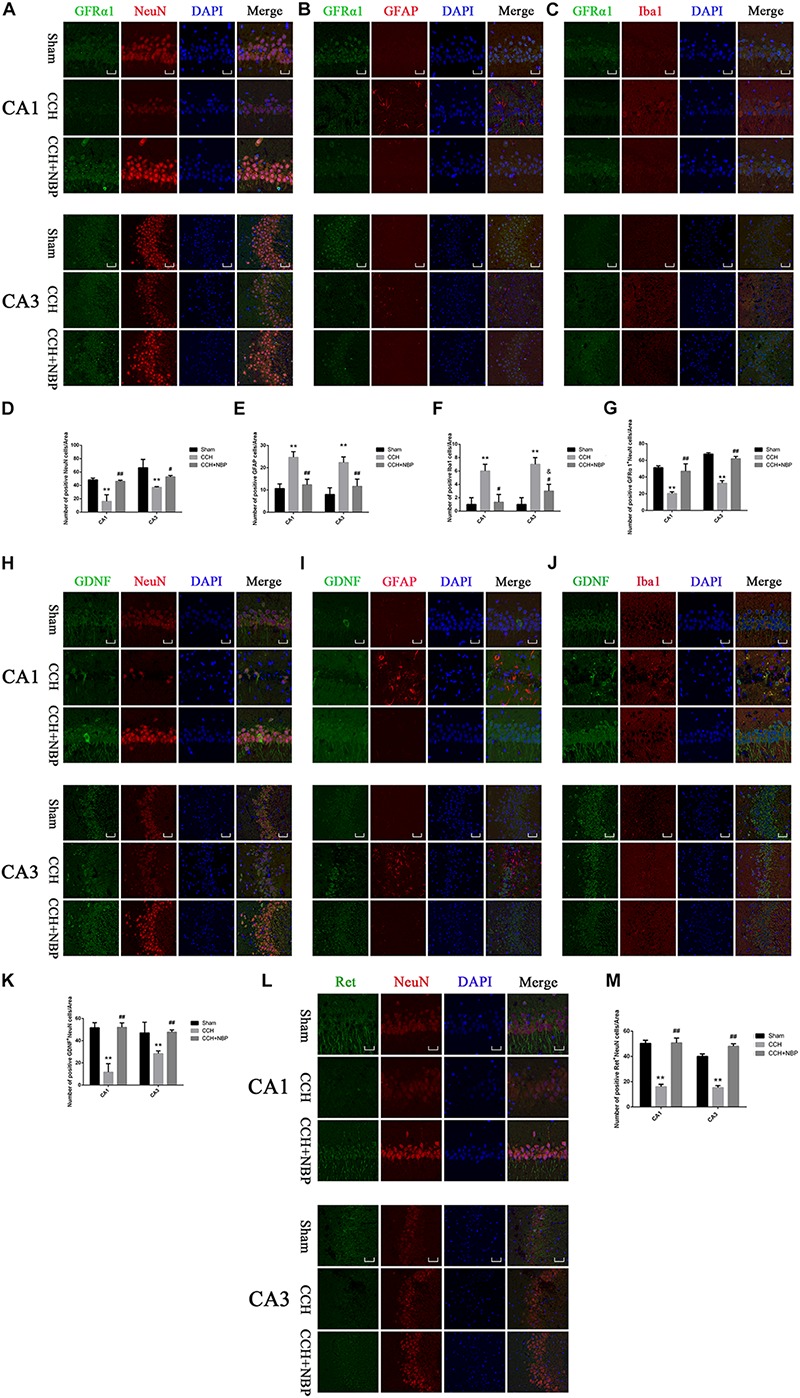
Glial cell line-derived neurotrophic factor/GDNF family receptor alpha-1/Receptor tyrosine kinase upregulation in neurons in different groups. Immunofluorescence staining of GFRα1 in the rat hippocampus at 8 weeks after BCCAO (CCH) in the different experimental groups. GFRα1 expression in neurons in the hippocampal CA1 and CA3 areas was determined using double immunofluorescence staining. **(A)** GFRα1 (green) upregulation after NBP treatment is observed in NeuN-positive neurons (red) in the hippocampus, but not in **(B)** GFAP-positive astrocytes (red) or **(C)** Iba1-positive microglia (red). **(D)** Quantitative analysis *(indicates changes of neurons in the studied areas (CA1, CA3) in response to CCH and NBP treatment. **(E)** Quantitative analysis indicates changes of astrocytes in the studied areas (CA1, CA3) in response to CCH and NBP treatment. **(F)** Quantitative analysis indicates changes of microglias in the studied areas (CA1, CA3) in response to CCH and NBP treatment. **(G)** Quantification of fluorescence images of the localization of GFRα1^+^NeuN-positive cells in the CA1 and CA3 areas. Immunofluorescence staining of GDNF in the hippocampus in different groups. Localization of GDNF expression in three neurons in the hippocampal CA1 and CA3 areas was determined using double immunofluorescence staining. **(H)** GDNF (green) upregulation after NBP treatment is observed in NeuN-positive neurons (red) in the hippocampus, but not **(I)** GFAP-positive astrocytes (red) or **(J)** Iba1-positive microglia (red). **(K)** Quantification of fluorescence images of the localization of GDNF^+^NeuN-positive cells in the CA1 and CA3 areas. **(L)** Immunofluorescence staining of Ret in the hippocampus in three groups. Localization of Ret (green) expression in neurons (red) in the hippocampal CA1 and CA3 areas was determined using double immunofluorescence staining. **(M)** Quantification of fluorescence images of the localization of Ret^+^NeuN-positive cells in the CA1 and CA3 areas. CA1 area, magnification 400×, scale bar = 25 μm, Area for quantitative analysis, 200 μm^2^; CA3 area, magnification 200×, scale bar = 50 μm, Area for quantitative analysis, 400 μm^2^. Images show representative results of 3 independent experiments. The values are mean ± SD. *n* = 3 animals per group. ^∗∗^*P* < 0.01, CCH group vs. sham group; ^#^*P* < 0.05, ^##^*P* < 0.01, CCH + NBP vs. CCH; ^&^*P* < 0.05, CCH + NBP vs. sham group. CCH: CCH 8w; CCH + NBP: CCH 8w + NBP.)*

The number of NeuN-positive cells in the CA1 and CA3 areas in CCH 8w rats was significantly decreased compared to that in sham rats, but significantly increased by NBP. In addition, there was no difference between the NBP-treated and the sham rats (*P* > 0.05) (Figures 5A,H). Quantitative analysis of data is shown in Figure 5D.

To determine the cellular localization of the GDNF/GFRα1/Ret protein in the hippocampus, co-immunofluorescence staining for GDNF/GFRα1/Ret and three neuron cells markers (NeuN; GFAP; Iba1) in brain sections of the three groups was performed. GFRα1/GDNF/Ret protein expression was localized within NeuN-positive cells (Figures 5A,H,L), but it was not observed in GFAP-positive astrocytes (Figures 5B,I) or Iba1-positive microglia (Figures 5C,J).

In addition, GFRα1^+^/GDNF^+^/Ret^+^-NeuN positive cells were downregulated in the hippocampus of CCH 8w rats but upregulated in those treated with NBP (Figures 5A,H,L). Quantitative analysis of data is shown in Figures 5G,K,M. *In situ* hybridization results showed that GDNF/GFRα1 were located in the neurons (Supplementary Figure S3g).

These findings suggest that upregulation of GDNF/GFRα1 binding to Ret in the hippocampus might be the mechanism-of-action of NBP treatment and is associated with neuronal survival in CCH rats.

#### NBP Reduced Hippocampus Glial Cell Activation in CCH Rats

The changes in GFAP and Iba1 immunofluorescent labeling are shown in Figure 5. Quantitative analysis demonstrated that the number of GFAP positive cells was increased in the CCH 8w group compared to the NBP-treated and sham groups (Figure 5E), as were the number of Iba1 positive cells (Figure 5F).

### *In vitro* Experiments

#### NBP Treatment Increased Primary Hippocampus Neuronal Viability and Reduced Apoptosis

After 7 days *in vitro*, the purity of the primary hippocampal neurons was tested (Supplementary Figure S4a). HIF-1α was increased, without severity of neuron death in OGD 1 h/R 48 h (Supplementary Figures S4c–e).

The effect of NBP alone (different concentrations) was tested *in vitro*. Thirteen different drug concentrations were screened, specifically, 10 μM, 20 μM, 30 μM, 40 μM, 50 μM, 60 μM, 70 μM, 80 μM, 90 μM, 100 μM, 125 μM, 150 μM, and 200 μM for 25 h (OGD 1 h/R 24 h). The results showed that when the concentration was below 60 μM, there were no damaging effects on neuronal survival (Supplementary Figure S4f). In addition, 10 μM, 30 μM, 50 μM, 60 μM were selected to evaluate the effect of NBP under OGD/R, the survival rate of neurons was increased effectively, especially at 60 μM (Supplementary Figure S4g).

**FIGURE 6:**
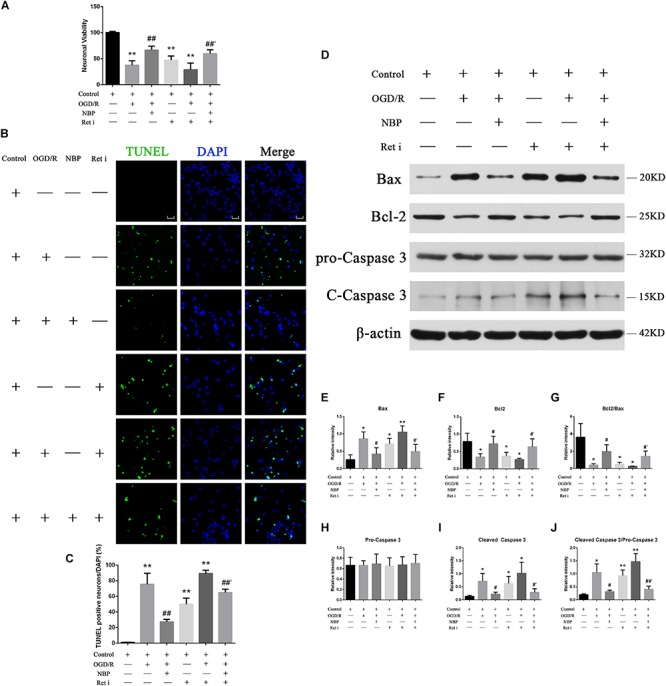
**(A)** CCK-8 results showed that OGD/R significantly increased neuron mortality (^∗∗^*P* < 0.01, compared to the control), and NBP treatment significantly increased cell viability (^##^*P* < 0.01, OGD/R + NBP (60 μM) vs. OGD/R). When the expression of Ret was inhibited, neuron viability was significantly decreased (^∗∗^*P* < 0.01, compared to the control), NBP treatment significantly increased cell viability (^##^′*P* < 0.01, OGD/R + Ret i + NBP (60 μM) vs. OGD/R + Ret i). *n* = 5 replicates per group. Ret i, Ret inhibitor; OGD/R, OGD 1 h/R 48 h. **(B)** TUNEL staining showed that OGD/R significantly increased neuronal apoptosis. NBP treatment significantly decreased the number of TUNEL-positive neurons. When the expression of Ret was inhibited, the number of TUNEL-positive neurons was significantly increased, and NBP treatment significantly decreased this number. Imaged at 400× magnification, scale bar = 25 μm. Area for quantitative analysis, 200 μm^2^. **(C)** Histogram showing quantitative analysis of TUNEL staining in different groups of primary hippocampal neurons. *n* = 3 texts per group. ^∗∗^*P* < 0.01, compared to the control; ^##^*P* < 0.01, OGD/R + NBP vs. OGD/R; ^##^′*P* < 0.01, OGD/R + Ret i + NBP vs. OGD/R + Ret i. Ret i, Ret inhibition. **(D)** NBP treatment reduced apoptosis of primary hippocampal neurons under OGD/R conditions by regulating Ret signaling. Western blotting showed OGD/R significantly increased neuron apoptosis (increased expression of Bax, cleaved caspase-3 and cleaved caspase-3/pro-caspase-3 ratio, decreased expression of Bcl-2 and a decreased Bcl-2/Bax ratio). NBP treatment significantly decreased apoptosis. When expression of Ret was inhibited, apoptosis was significantly increased. NBP treatment significantly decreased neuron apoptosis. **(E)** Histogram showing quantitative analysis of the relative intensity of Bax. **(F)** Histogram showing quantitative analysis of the relative intensity of Bcl-2. **(G)** Histogram showing quantitative analysis of the Bcl-2/Bax ratio. **(H)** Histogram showing quantitative analysis of the relative intensity of pro-caspase 3. **(I)** Histogram showing quantitative analysis of the relative intensity of cleaved caspase-3. **(J)** Histogram for quantitative analysis of cleaved-caspase-3/pro-caspase-3. *n* = 3 replicates per group. ^*^P < 0.05, ^∗∗^*P* < 0.01, compared to the control; ^#^*P* < 0.05, OGD/R + NBP vs. OGD/R; ^#^′*P* < 0.05, ^##^′*P* < 0.01, OGD/R + Ret i + NBP vs. OGD/R + Ret i. Ret i, Ret inhibition.

Oxygen-glucose deprivation 1 h/R 48 h decreased the survival rate of neurons to 47.8%, while treatment with NBP (60 μM) increased neuron survival to 74.9% (Figure 6A). OGD 1 h/R 48 h treatment increased the number TUNEL-positive neurons (Figures 6B,C) compared to those in control and NBP-treated (60 μM) neurons.

Western blotting results showed that OGD 1 h/R 48 h led to increased expression of Bax and cleaved caspase-3, as well as an increased cleaved caspase-3/pro-caspase-3 ratio. It also led to decreased expression of Bcl-2 and a decreased Bcl-2/Bax ratio. Treatment with NBP reversed these effects (Figure 6D). A quantitative analysis of the data is shown in Figures 6E–J.

#### Expression Levels of GDNF/GFRα1/Ret and p-AKT/p-ERK1/2 in Hippocampal Neurons Following OGD/R or NBP Treatment

**FIGURE 7:**
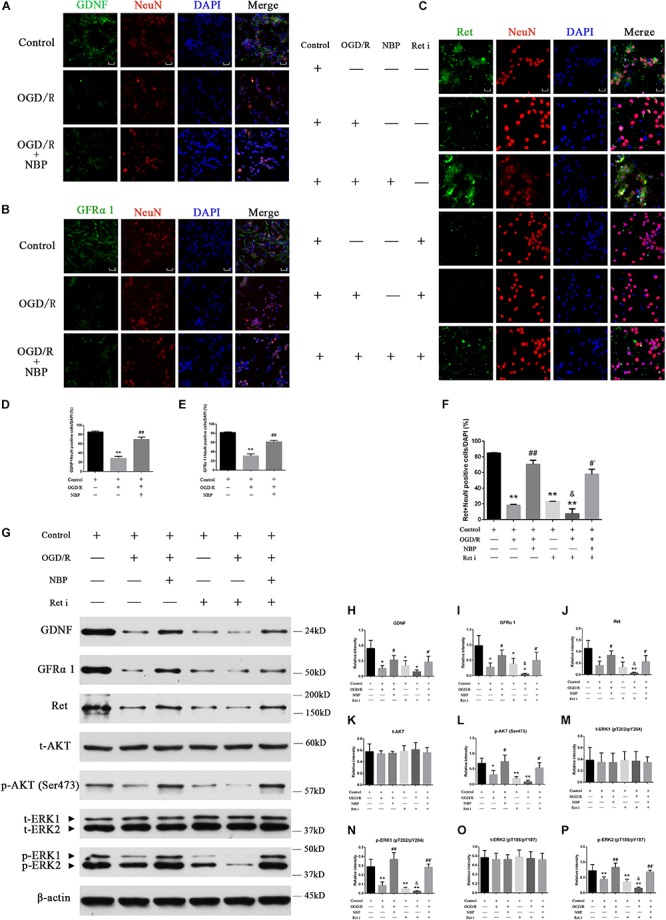
Dl-3-n-butylphthalide activates GDNF/GFRα1/Ret and downstream p-AKT/p-ERK1/2 signaling, protecting against hippocampal neuron apoptosis in an OGD/R model. **(A)** Immunofluorescence shows that OGD/R significantly decreased positive GDNF^+^ neurons, while NBP treatment increased positive GDNF^+^ neurons. 400× magnification, scale bar = 25 μm. Area for quantitative analysis, 200 μm^2^. **(B)** Immunofluorescence shows that OGD/R significantly decreased positive GFRα1^+^ neurons, while NBP treatment increased positive GFRα1^+^ neurons. 400× magnification, scale bar = 25 μm. Area for quantitative (analysis, 200 μm^2^. **(C)** Immunofluorescence shows that OGD/R significantly decreased positive Ret^+^ neurons, while NBP treatment increased positive Ret^+^ neurons. Ret inhibitor also decreased positive Ret^+^ neurons, while NBP treatment increased positive Ret^+^ neurons. 400× magnification, scale bar = 25 μm. Area for quantitative analysis, 200 μm^2^. **(D)** Histogram showing quantitative analysis of positive GDNF^+^ neurons. *n* = 3 replicates per group. ^∗∗^*P* < 0.01, OGD/R vs. control; ^##^*P* < 0.01, OGD/R + NBP vs. OGD/R. **(E)** Histogram showing quantitative analysis of positive GFRα1^+^ neurons. *n* = 3 replicates per group. ^∗∗^*P* < 0.01, OGD/R vs. control; ^##^*P* < 0.01, OGD/R + NBP vs. OGD/R. **(F)** Histogram showing quantitative analysis of positive Ret^+^ neurons. *n* = 3 replicates per group. ^∗∗^*P* < 0.01, vs. control; ^##^*P* < 0.01, OGD/R + NBP vs. OGD/R; ^&^*P* < 0.05, OGD/R + Ret i vs. OGD/R; ^#^′*P* < 0.05, OGD/R + Ret i + NBP vs. OGD/R + Ret i. Ret i, Ret inhibition. **(G)** Western blotting showed that OGD/R significantly decreased the expression of GDNF/GFRα1/Ret/p-AKT/p-ERK1/2, while NBP treatment significantly increased the expression of GDNF/GFRα1/Ret/p-AKT/p-ERK1/2. When the expression of Ret was inhibited, GDNF/GFRα1 and p-AKT/p-ERK1/2 were also significantly decreased, but NBP treatment significantly increased the expression of GDNF/GFRα1 and p-AKT/p-ERK1/2. **(H)** Histogram showing quantitative analysis of the relative intensity of GDNF. **(I)** Histogram showing quantitative analysis of the relative intensity of GFRα 1. **(J)** Histogram showing quantitative analysis of the relative intensity of Ret. **(K)** Histogram showing quantitative analysis of the relative intensity of t-AKT. **(L)** Histogram showing quantitative analysis of the relative intensity of p-AKT (Ser473). **(M)** Histogram showing quantitative analysis of the relative intensity of t-ERK1 (pT202/pY204). **(N)** Histogram showing quantitative analysis of the relative intensity of p-ERK1 (pT202/pY204). **(O)** Histogram showing quantitative analysis of the relative intensity of t-ERK2 (pT185/pY187). **(P)** Histogram showing quantitative analysis of the relative intensity of p-ERK2 (pT185/pY187). *n* = 3 replicates per group. ^*^P < 0.05, ^∗∗^*P* < 0.01, vs. control; ^#^*P* < 0.05, ^##^*P* < 0.01, OGD/R + NBP vs. OGD/R; ^&^*P* < 0.05, OGD/R + Ret i vs. OGD/R; ^#^′*P* < 0.05, ^##^′*P* < 0.01, OGD/R + Ret i + NBP vs. OGD/R + Ret i. OGD/R, OGD 1 h/R 48 h, Ret i, Ret inhibition.)

Compared with control or NBP treatment, OGD 1 h/R 48 h significantly decreased positive GDNF^+^/GFRα1^+^/Ret^+^ neurons (Figures 7A–C). Quantitative analysis of data is shown in Figures 7D–F. Western blotting results showed that NBP given alone *in vitro* model did not significantly affect the expression of GDNF/GFRα1/Ret and p-AKT/p-ERK1/2 (Supplementary Figures S4J,k). But OGD 1 h/R 48 h led to downregulation of the expression of GDNF/GFRα1/Ret and p-AKT/p-ERK1/2; NBP treatment can significantly increase expression of GDNF/GFRα1/Ret and p-AKT/p-ERK1/2 (Figure 7G). Quantitative analysis of the data is shown in Figures 7H–P.

#### Inhibiting Ret Increases Apoptosis, While NBP Regulates Ret Expression and Reduces Neuronal Apoptosis in the OGD/R Model

To probe for a direct link between GDNF/GFRα1/Ret and neuronal apoptosis in CCH, we used an OGD 1 h/R 48 h model and Ret inhibition in hippocampal neurons. Neurons were treated with normal control, OGD 1 h/R 48 h (OGD/R), OGD 1 h/R 48 h + NBP (NBP), Ret inhibitor (Ret i), OGD 1 h/R 48 h + Ret inhibitor (OGD/R + Ret i), and OGD 1 h/R 48 h + Ret inhibitor + NBP (OGD/R + Ret i + NBP).

When the expression of Ret was inhibited, neuronal death was significantly increased. CCK-8 results showed that neuron survival decreased (Figure 6A), the number of TUNEL positive cells increased (Figures 6B,C), and the expression of apoptotic proteins increased (Figure 6D). Under conditions of OGD/R, NBP improved neuron survival, even in combination with Ret i (Figure 6A). Expression of apoptotic proteins was also relatively decreased in NBP-treated rats (Figure 6D). Quantitative analysis of the data is shown in Figures 6E–J.

#### NBP Protects Neurons by Regulating GDNF/GFRα1/Ret Signaling in OGD/R

The Ret i inhibits the expression of the Ret protein in normal hippocampal neurons. Upstream activators of Ret, including GDNF and GFRα1, and its downstream effectors of Ret, including p-AKT and p-ERK1/2, were also inhibited (Figure 7G). Pre-treatment with NBP increased the expression of GDNF/GFRα1/Ret, p-AKT and p-ERK1/2, even when combined with Ret i (Figure 7G). Quantitative analysis of the data is shown in Figures 7H–P.

## Discussion

A comprehensive understanding of the status of a disease cannot be obtained from genomic studies alone. This study used a new approach, using an antibody microarray, to identify potential therapeutic targets associated with CCH induced hippocampal neuron apoptosis and NBP treatment. Antibody microarrays are high-throughput tools, with lower sample volume and antibody concentration requirements and higher format versatility. Its applications include disease biomarker discovery for drug response, diagnosis, characterization of protein pathways (Sanchez-Carbayo, 2006; Jaeger et al., 2016; Li et al., 2019). Through antibody microarray detection, we identified the expression of GDNF, GFRα1, and Ret was decreased in the hippocampal tissue of the CCH 8w; however, NBP produced an inverse effect. At present, there are no researches assessing the correlation between NBP and the GDNF/GFRα1/Ret signaling axis. This current study provides insight into the potential protective effects of NBP treatment against CCH induced hippocampal neuron apoptosis by regulation of GDNF/GFRα1/Ret signaling, resulting in improved cognitive function.

### BCCAO Lead to Hypoperfusion and Cognitive Impairment, Accompany With Hippocampal Neuron Apoptosis

Lower CBF is predictive of a higher future risk of preclinical dementia (Hays et al., 2016; Raz et al., 2016). Permanent BCCAO is a classical experimental animal model for studying mechanisms of cognitive impairment underlying CCH (Du et al., 2017; Xiong et al., 2017; Zhang et al., 2017). At 8 weeks after BCCAO, concomitant cortical hypoperfusion and cognitive impairment were observed. HIF-1α is a primary transcriptional mediator of the hypoxic response and a master regulator of O_2_ homeostasis (Yang et al., 2017). The levels of HIF-1α were significantly increased at BCCAO 8w in our study, indicating that a hypoxic state remained. Therefore, BCCAO 8w/CCH 8w was selected as the successful establishment of the CCH induced VD model for further study.

Many researches claimed that cognitive damage and pathological changes that occur due to CCH can be attenuated by improvement of the CBF. However, although CBF gradually returned to its pre-occlusion level, neuropathological changes did not improve and actually deteriorated with the passage of time. A cascade effect may occur once an individual has experienced CCH for a period of time and that the methods used to recover CBF are effective only at earlier time points (Zou et al., 2018). CCH induces a compensatory mechanism to maintain optimal CBF, but this mechanism is limited and cannot prevent neuronal loss or cognitive impairment (Jing et al., 2015). In addition, the effect of NBP in improving CBF and is also limited (Li et al., 2019).

A long-period hypoxia and HIF-1α expression in adult organisms may contribute to angiogenesis (Semenza, 2003; Greijer et al., 2005). Compared to the sham rats, the number of CD34 positive cells were increased in the cortex of CCH 8w and NBP-treated groups, indicating that angiogenesis occurred with effect of NBP, and at a later time point in CCH. Further, the number of CD34 positive cells was the same in the three groups and hippocampal CBF completely recovered to the pre-operative levels, the hippocampus is largely supplied by the posterior cerebral artery, and receives only a minor contribution from the anterior choroidal artery from the internal carotid artery (Kirschen et al., 2018). In addition, thickened vertebral arteries may provide a better blood supply (Xiong et al., 2017; Li et al., 2019). Although CBF recovered in the later stage of CCH to a certain degree, hippocampal neurons apoptosis in CA1 and CA3 areas was irreversible. At the same time point, learning and memory was significantly impaired. Further elucidation of the basic molecular mechanisms underlying the hippocampus neuronal apoptosis in CCH will be invaluable to the development of new therapeutic approaches.

### GDNF/GFRα1/Ret Signaling and Neuron Survival

Glial cell line-derived neurotrophic factor is one of the members of the four ligands in the GDNF family, which belong to the transforming growth factor-β superfamily (Drinkut et al., 2016). GDNF signaling is mediated by two-component receptor consisting of GFRα1 and the transmembrane receptor tyrosine kinase Ret. GDNF dimers bind preferentially to GFRα1 with high affinity. In addition, GDNF may use the α1-NCAM dependent signaling pathway instead of the Ret-dependent pathway. Once activated, the resulting complex recruits Ret and sends signals through the brain by forming a multicomponent receptor complex, composed of the glycosyl-phosphatidyl inositol-anchored receptor GFRα1 and Ret. This leads to its activation at specific cytoplasmic tyrosine residues, thus initiating a number of downstream intracellular pathways (Sariola and Saarma, 2003; Curcio et al., 2015; Kramer and Liss, 2015). Ret can activate various signaling pathways, such as ERK, PI3K/AKT, p38 MAPK, and JNK pathways (Ichihara et al., 2004; Yue et al., 2017). These pathways have been demonstrated to play an important role in regulating cell proliferation, apoptosis, and survival in various systems.

Deficiency of GDNF receptor GFRα1 or Ret in neurons, results in neuronal death in AD and PD (Konishi et al., 2014; Kramer and Liss, 2015; Requejo et al., 2018). The absence of Ret completely abolished the neuroprotective and regenerative effects of GDNF on the midbrain dopaminergic system. This establishes Ret signaling as absolutely required for GDNF to prevent and compensate for dopaminergic system degeneration, which suggests Ret activation is the primary target of GDNF therapy in PD (Drinkut et al., 2016). In cultured hippocampal neurons, brain ischemia downregulates the neuroprotective GDNF-Ret signaling by a calpain-dependent mechanism. Preserving Ret receptors may be a good strategy to increase the endogenous neuroprotective mechanisms (Curcio et al., 2015). It suggests that the main goal of clinical trials using GDNF and related substances should be to activate the Ret receptor. Although transient ischemic injury upregulates GDNF expression in the injured region of the brain, this is less likely to result in an increase in neuroprotection (Curcio et al., 2015). CCH is a process in the chronic ischemic category; therefore, under conditions of CCH, the downregulation of Ret affects GFRα1 and the GDNF/GFRα1 complex, which also affects downstream effectors of Ret, including p-AKT and p-ERK1/2. This eventually leads to delayed neuron death. Based on what has been discussed above, activating GDNF/GFRα1/Ret signaling may be a good strategy to increase endogenous neuroprotective mechanisms in CCH-induced VD (Curcio et al., 2015; Ibanez and Andressoo, 2017).

### Potential Molecular Contribution of NBP in CCH Induced VD

The complex etiology of CCH means that multifunctional agents may be beneficial for the treatment of this disease. In a previous study where NBP was tested in CCH animals, NBP could reduce cognitive impairment induced by CCH, but the molecular mechanisms underlying the therapeutic effect were different as the study investigated the therapeutic effects of NBP on downregulation of the amyloid precursor protein Aβ40, MMP-2 and MMP-9 proteins in cortex and hippocampus (Wei et al., 2012). An amelioration in delayed neuronal death occurring after CCH might be a vital therapeutic target to improve the long-term outcome of VD. In this current study, CBF in the bilateral cortex in NBP-treated rats recovered compared to the pre-operation stage with a decrease in HIF-1α levels, with cognitive improvement. However, the neuroprotective effect of NBP ameliorates hippocampal neuron apoptosis is also noteworthy to study in CCH models.

Neurotrophic factors are good therapeutic candidates for neurodegenerative diseases. GDNF is a diffusible peptide, involved in neuronal differentiation and survival, and has been identified as potential biomarker in AD in an unbiased proteomic assay (Ray et al., 2007), in amyotrophic lateral sclerosis (Stanga et al., 2018) and is the most potent dopaminergic factor described for the treatment of PD (Garbayo et al., 2016). In early acute hypoxia, GDNF counteracts acute hypoxic damage to hippocampal neural network function *in vitro* (Shishkina et al., 2018).

In the course of a long-term hypoxia attack, GDNF/GFRα1/Ret signaling was downregulated and not the NCAM dependent signaling pathway, which may inform the development of potential therapeutic strategies for CCH induced VD through upregulation of GDNF/GFRα1/Ret expression. However, previous attempts to move GDNF into clinical practice have been only moderately successful. One of the factors limiting the effectiveness of GDNF therapies is its short biological half-life due to its labile nature. Furthermore, the factor does not cross the blood brain barrier (BBB) and often has serious side effects when administered systemically, necessitating an effective drug delivery strategy to reach the brain. Therefore, in order to use GDNF effectively as a therapeutic agent, it is essential to develop a safe and effective brain delivery system (Garbayo et al., 2016). Gene and cell therapy have experienced moderate success, but are also complex and expensive. Therefore, the development of small-molecule drugs that can pass the BBB is a promising prospect.

Racemic NBP is a multi-target neuroprotective agent and is a chiral compound containing L- and D-isomers. It is also a small molecule, and, with HP-β-CD as the carrier, can easily pass through the BBB (Diao et al., 2015). Although it has previously been studied, new molecular targets of NBP are still worth exploring.

**FIGURE 8:**
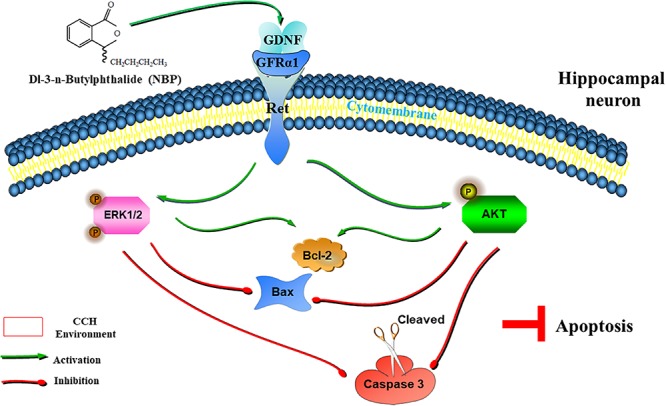
Graphical Abstract of the proposed anti-apoptotic signaling pathways triggered by NBP in hippocampus neurons. NBP regulates GDNF/GFRα1/Ret signaling in a CCH environment, activates downstream p-AKT(Ser473) and p-ERK1(pT202/pY204)/ERK2(pT185/pY187) pathways, upregulates the expression of Bcl-2, and downregulates the expression of Bax and cleaved caspase-3, which ultimately block apoptosis.

With a combination of *in vivo* and *in vitro* experiments, we show that NBP promotes the upregulation of Ret. It then upregulated GDNF/GFRα1 and caused downstream effector pathways, specifically AKT and ERK1/2 pathways, to become activated and reduce hippocampal apoptosis, and improve cognitive function in CCH (Figure 8). In addition, activation of astrocytes and microglia was significantly reduced in the NBP rats, consistent with our previous study (Xiong et al., 2017). GDNF family ligands are able to regulate microglial function and may play a role in the suppression of microglial activation, as microglia are the target cells of members of the GDNF family (Rickert et al., 2014). Reduction of activated glia may be associated with improved collateral circulation, or the reduced delayed neuronal apoptosis, by NBP. Some researchers indicated that BCCAO might not effectively represent a clinical VD model, due to the limitation of CCH process induced by BCCAO. Therefore, further experiments including more experimental models of VD/more behavioral tests, are needed to support, and to determine the deeper molecular mechanism of CCH and targets of NBP.

## Conclusion

The, GDNF/GFRα1/Ret signaling might be a potential therapeutic target in CCH. NBP treatment may be a good strategy to mediate cognitive improvement. It may also help reduce hippocampal neuron apoptosis by regulating neuroprotective mechanisms of GDNF/GFRα1/Ret signaling.

## Data Availability

The raw data supporting the conclusions of this manuscript will be made available by the authors, without undue reservation, to any qualified researcher (Supplementary Table S1).

## Ethics Statement

All animal protocols were approved by the institutional animal care committee of the experimental animal management center of Jinan University (Guangzhou, China) and conformed to internationally accepted ethical standards (Guide for the Care and Use of Laboratory Animals. United States NIH Publication 86-23, revised 1985), ensuring humane and proper care of research animals.

## Author Contributions

WL, LH, and DW designed the research and prepared the manuscript. WL and XX performed the BCCAO rat model and measured the water maze experiments. WL, JYL, and JXL performed the experiments with magnetic resonance imaging. WL and DW performed the primary neuronal cell culture and related experiments and performed all the experiments related to histology, immunohistochemistry, and Western blot analyses. WL, KS, and JXL collected and analyzed the data.

## Conflict of Interest Statement

The authors declare that the research was conducted in the absence of any commercial or financial relationships that could be construed as a potential conflict of interest.

## Footnotes

1 https://string-db.org/cgi/input.pl

2 https://blast.ncbi.nlm.nih.gov/Blast.cgi

3 https://www.ncbi.nlm.nih.gov/gene/?term=

## Supplementary Material

FIGURE S1 **(a)** Structure of NBP. Its chemical name is (±) 3-butyl-3H-2-benzofuran-1-one (chemical formula: C_12_H_14_O_2_; molecular weight: 190.24). **(b)** A representative HE stained section showing selected areas in the cortex and hippocampus (CA1, CA3 and DG area) for evaluation. **(c,d)** Changes in CBF after BCCAO CBF was measured in the cortex and hippocampus of rats pre-occlusion, immediately following BCCAO, and 1, 2, 4, and 8 weeks after BCCAO using ASL. In cortical areas, CBF was consistently lower after BCCAO than before, while CBF was restored in the hippocampus at the 8th week after BCCAO. Histograms show quantitative results of the CBF at different time points after BCCAO. CBF in the bilateral cortex was significantly decreased upon BCCAO, as well as the 1st, 2nd, 4th, and 8th weeks after BCCAO, compared to CBF pre-occlusion **(c)**. CBF in the bilateral hippocampus was significantly decreased upon BCCAO, as well as the 1st, 2nd, and 4th weeks after BCCAO, compared to CBF pre-occlusion, but was increased in the 8th week (*P* > 0.05) **(d)**. ^*^*P* < 0.05, ^∗∗^*P* < 0.01, compared to pre-BCCAO; ^#^*P* < 0.05, ^##^*P* < 0.01, BCCAO 8w vs. BCCAO 4w. L, left side; R, right side. Changes in learning and memory after BCCAO. **(e)**We investigated whether the BCCAO model used in the study would induce cognitive impairment. Rats were trained to perform the MWM test. After surgery, the EL was significantly prolonged at the 2nd, 4th, and 8th week after BCCAO (^*^*P* < 0.05, ^∗∗^*P* < 0.01, CCH 2w vs. sham; ^#^*P* < 0.05, ^##^*P* < 0.01, CCH 4w vs. sham; ^&&^*P* < 0.01, CCH 8w vs. sham). **(f)** On the 6th day, rats were allowed to navigate the water for 60 s. Quantitative data showed that the frequency of crossing the original platform was decreased 2, 4, and 8 weeks after BCCAO (^*^*P* < 0.05 compared to sham). Morphological changes in pyramidal cells and delayed neuronal death in BCCAO groups. Morphological changes in pyramidal cells were found in the CA1 and CA3 areas of the CCH 8w group. They also occurred in the CA1 area of CCH 4w group, but were not found in the CCH 2w or sham groups **(g)**. Delayed neuronal death was observed in the CA1 and CA3 areas of the CCH 8w group, as well as in the CA1 area of the CCH 4w group, but it has not been observed in the CCH 2w or sham groups. Neurons in those areas denoted hyperchromatism, shown with pyknosis and shaped into diamond and triangle **(h)**. CA1, CA3, and DG areas, magnification 200×, scale bar = 50 μm.

FIGURE S2 Changes of the CD34 positive cells. Immunofluorescence labeling showing the number of CD34 positive cells in the cortex, CA1, CA3, and DG in the three groups. **(a–d)** Quantitative analysis. Cortex area, magnification 400×; CA1 area, magnification 400×, scale bar = 25 μm. CA3 and DG areas, magnification 200×, scale bar = 50 μm. The values are mean ± SD. *n* = 3 animals per group. Cortex: ^∗∗^ < 0.01, CCH group vs. sham group; ^&&^ < 0.01, CCH + NBP group vs. sham group. CA1, CA3, DG: No statistically significant differences among the three groups. CCH: CCH 8w; CCH + NBP: CCH 8w + NBP.

FIGURE S3 Differentially expressed proteins between CCH 8w and sham groups, and biological function analysis. **(a)** Clustering analysis showed six DEPs (GFRα1, GFRα2, E-Selection, Fractalkine, and I-3, Insulin) were differentially expressed in the hippocampus between CCH 8w and sham groups (*p* < 0.01). **(b)** Conservative analysis of six DEPs. The conservation of the six DEPs between *Homo sapiens* and *Rattus norvegicus* was analyzed. The results showed that GFRα1 was the most conserved DEP (Query cover = 100%; *E*-value = 0.0; Align identities score = 90.81%). **(c)** GO enrichment of DEPs: BP. **(d)** GO enrichment of DEPs: CC. **(e)** GO enrichment of DEPs: MF. **(f)** KEGG enrichment analysis of DEPs. **(g)** Localization of GFRα 1 and GDNF mRNA as shown by *in situ* hybridization upon NBP treatment. magnification 400×, scale bar = 25 μm.

FIGURE S4 **(a)** Purity test of primary hippocampal neurons. 400× magnification. **(b,c)** Changes in neuronal viability after OGD/R Neuron viability was measured in primary hippocampal neuron cultures under the following conditions: normal, OGD 0.5 h, OGD 1 h, OGD 1.5 h, OGD 2 h, as well as OGD 1 h/R 0 h, 0.5 h, 3 h, 6 h, 12 h, 24 h, 48 h, 72 h, and 96 h using CCK-8. After OGD or OGD 1 h/R, the viability of neurons had decreased to different degrees **(b)**, ^∗∗^*P* < 0.01 compared to sham, ^#^*P* < 0.05, ^##^*P* < 0.01 compared to OGD 0.5 h; **(c)**, ^∗∗^*P* < 0.01 compared to sham, ^#^*P* < 0.05, ^##^*P* < 0.01 compared to OGD 1 h/R 48 h. **(d,e)** Expression of HIF-1α after OGD/R in primary hippocampal neurons. Western blotting results showed that after OGD 1 h/R 6 h, OGD 1 h/R 12 h, OGD 1 h/R 24 h, OGD 1 h/R 48 h, HIF-1α expression was significantly increased. *n* = 3 per experiments. ^*^*P* < 0.05, compared to control. **(f–i)** Experimental screening of drug and reagent concentrations In order to select the optimal concentration of NBP to treat neurons, we screened 13 different drug concentrations, specifically, 10, 20, 30, 40, 50, 60, 70, 80, 90, 100, 125, 150, and 200 μM for 24 h. The results showed that when the drug concentration was below 60 μM, there were no damaging effects on neuronal survival **(f)**. ^*^*P* < 0.05, ^∗∗^*P* < 0.01 compared with normal control. The effect of various concentrations of NBP (10, 30, 50, and 60 μM) on OGD/R (OGD 1 h/R 48 h)-induced neuronal cell damage **(g)**. ^∗∗^*P* < 0.01 compared with normal control, ^##^*P* < 0.01 compared with OGD/R, ^&&^*P* < 0.01 compared with 10 μM, ^&^′*P* < 0.05 compared with 30 μM. In order to select the optimal concentration of Ret inhibitor, we set up 8 concentrations, 60, 80, 100, 1, 2, 3, 4, and 5 μM for 6 h. The results showed that treatment with 60 nM Ret inhibitor for 6 h had a significant adverse effect on neuronal survival. At a concentration of 5 μM for 6 h, the expression of Ret could be significantly inhibited **(h)**. ^*^*P* < 0.05, ^∗∗^*P* < 0.01 compared to control, ^#^*P* < 0.05 compared to 1 μM. Western blotting revealed that as the reagent concentration increased, the Ret protein was significantly inhibited **(i)**. **(j,k)** Expression of GDNF/GFRα1/Ret and downstream signal proteins in primary hippocampal neurons after OGD/R. Western blotting results showed that NBP given alone *in vitro* model, GDNF/GFRα1/Ret and downstream signal proteins, including p-AKT and p-ERK1/2, were not significantly changed **(j)**. Quantitative analysis results shown in **(k)**. *n* = 3 per experiments.

FIGURE S5 **(a)** Full western blots from *in vivo* experiments. Proteins were extracted with RIPA from sham, CCH, and NBP groups. Blots were probed with the indicated antibodies. The levels of the protein of interest were normalized to β-actin for every sample. These indicators included GDNF, GFRα1, Ret, NCAM, HIF-1α, pro-caspase 3, cleaved caspase 3, Bcl-2, Bax, t-AKT, p-AKT (Ser473), t-ERK1 (pT202/pY204)/ERK2 (pT185/pY187), p-ERK1 (pT202/pY204)/ERK2 (pT185/pY187). CCH: CCH 8w; NBP: CCH 8w + NBP. **(b)** Full western blots from *in vitro* experiments. Proteins were extracted with RIPA from seven groups, including the control, OGD/R, OGD/R + NBP, Ret i, OGD/R + Ret i, OGD/R + Ret i + NBP, and control + NBP. Blots were probed with the indicated antibodies. The levels of the protein of interest were normalized to β-actin for every sample. These indicators included GDNF, GFRα1, Ret, HIF-1α, pro-caspase 3, cleaved caspase 3, Bcl-2, Bax, t-AKT, p-AKT (Ser473), t-ERK1 (pT202/pY204)/ERK2 (pT185/pY187), p- ERK1 (pT202/pY204)/ERK2 (pT185/pY187). **(b1)** Ret. **(b2)** HIF-1α. **(b3)** First trial. (1 control, 2 OGD/R, 3 OGD/R + NBP, 4 Ret i, 5 OGD/R + Ret i, 6 OGD/R + Ret i + NBP, 7 control + NBP, 8 OGD/R.) **(b4)** Second trial. (1′ control, 2′ OGD/R, 3′ OGD/R + NBP, 4′ Ret i, 5′ OGD/R + Ret i, 6′ OGD/R + Ret i + NBP, 7′ control + NBP, 8′ OGD/R.) **(b5)** Third trial. (1^″^ control, 2^″^ OGD/R, 3^″^ OGD/R + NBP, 4^″^ Ret i, 5^″^ OGD/R + Ret i, 6^″^ OGD/R + Ret i + NBP, 7^″^ control + NBP, 8^″^ OGD/R).

TABLE S1 All data from the antibody microarray analyses.

## Abbreviations

3D ASL: three dimensional arterial spin labeling
ACTH: proopiomelanocortin (POMC)
AD: Alzheimer’s disease
BCCAO: bilateral common carotid artery occlusion (BCCAO)
BP: biological processes
CBF: cerebral blood flow
CC: cellular components
CCH: chronic cerebral hypoperfusion
DEPs: differentially expressed proteins
DMEM: dulbecco’s minimum essential medium
EL: escape latency
GDNF: glial cell line-derived neurotrophic factor
GFR α 1: glial cell line-derived neurotrophic factor family receptor alpha-1
GFR α 2: glial cell line-derived neurotrophic factor family receptor alpha-2
GO: gene ontology
IL-3: Interleukin-3
KEGG: kyoto encyclopedia of genes and genomes
MF: molecular function
MRI: magnetic resonance imaging
MWM: morris water maze
NBP: Dl-3-N-butylphthalide
NCAM: neural cell adhesion molecule
OGD/R: oxygen-glucose deprivation (OGD)/refusion (R)
PD: Parkinson’s disease
PPI: protein–protein interaction
Ret i: ret kinase inhibitor
Ret: receptor tyrosine kinase
TEM: transmission electron microscope
TIMP-1: tissue inhibitors of metalloproteinase-1
VD: vascular dementia.
