# Supplementary figures and images for: Dl-3-n-Butylphthalide Reduces Cognitive Impairment Induced by Chronic Cerebral Hypoperfusion Through GDNF/GFRα1/Ret Signaling Preventing Hippocampal Neuron Apoptosis
Source: Front Cell Neurosci. 2019 Aug 13;13:351. doi: 10.3389/fncel.2019.00351 (PMC6701226; doi:10.3389/fncel.2019.00351)

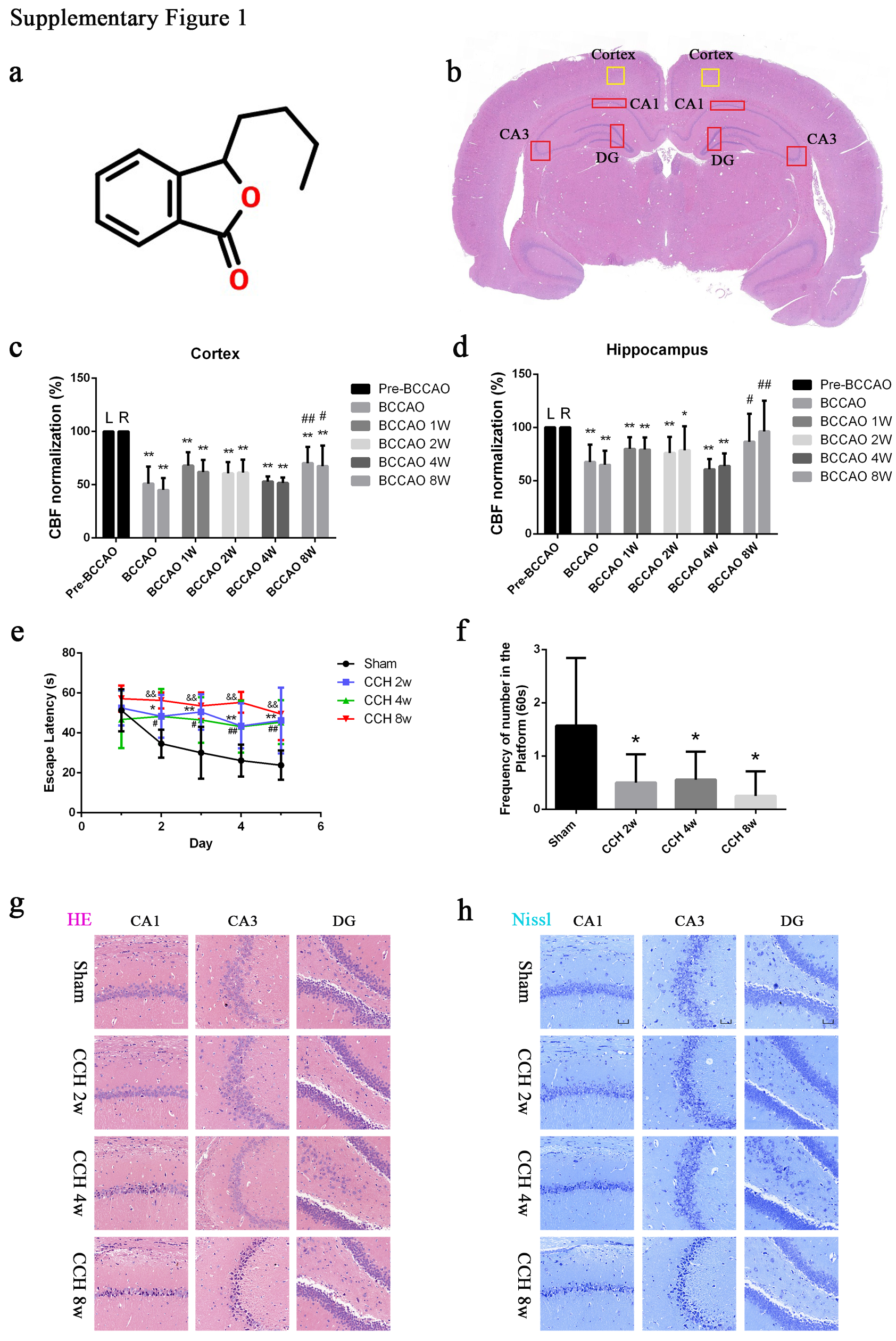

Supplement: FIGURE S1 — (a) Structure of NBP. Its chemical name is (±) 3-butyl-3H-2-benzofuran-1-one (chemical formula: C12H14O2; molecular weight: 190.24). (b) A representative HE stained section showing selected areas in the cortex and hippocampus (CA1, CA3 and DG area) for evaluation. (c,d) Changes in CBF after BCCAO CBF was measured in the cortex and hippocampus of rats pre-occlusion, immediately following BCCAO, and 1, 2, 4, and 8 weeks after BCCAO using ASL. In cortical areas, CBF was consistently lower after BCCAO than before, while CBF was restored in the hippocampus at the 8th week after BCCAO. Histograms show quantitative results of the CBF at different time points after BCCAO. CBF in the bilateral cortex was significantly decreased upon BCCAO, as well as the 1st, 2nd, 4th, and 8th weeks after BCCAO, compared to CBF pre-occlusion (c). CBF in the bilateral hippocampus was significantly decreased upon BCCAO, as well as the 1st, 2nd, and 4th weeks after BCCAO, compared to CBF pre-occlusion, but was increased in the 8th week (P > 0.05) (d). *P < 0.05, ∗∗P < 0.01, compared to pre-BCCAO; #P < 0.05, ##P < 0.01, BCCAO 8w vs. BCCAO 4w. L, left side; R, right side. Changes in learning and memory after BCCAO. (e)We investigated whether the BCCAO model used in the study would induce cognitive impairment. Rats were trained to perform the MWM test. After surgery, the EL was significantly prolonged at the 2nd, 4th, and 8th week after BCCAO (*P < 0.05, ∗∗P < 0.01, CCH 2w vs. sham; #P < 0.05, ##P < 0.01, CCH 4w vs. sham; &&P < 0.01, CCH 8w vs. sham). (f) On the 6th day, rats were allowed to navigate the water for 60 s. Quantitative data showed that the frequency of crossing the original platform was decreased 2, 4, and 8 weeks after BCCAO (*P < 0.05 compared to sham). Morphological changes in pyramidal cells and delayed neuronal death in BCCAO groups. Morphological changes in pyramidal cells were found in the CA1 and CA3 areas of the CCH 8w group. They also occurred in the CA1 area of CCH 4w grou [file Image_1.TIF]

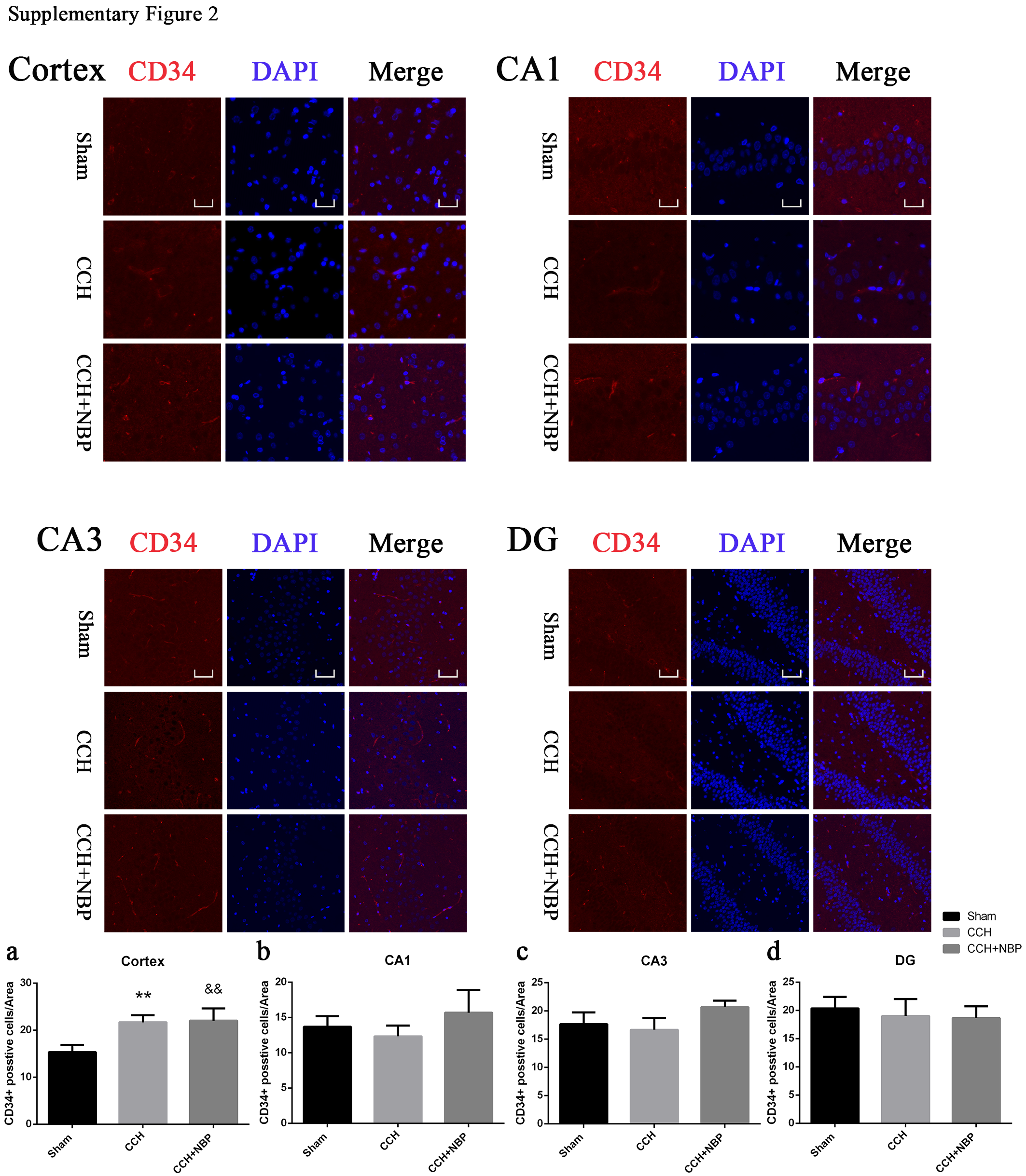

Supplement: FIGURE S2 — Changes of the CD34 positive cells. Immunofluorescence labeling showing the number of CD34 positive cells in the cortex, CA1, CA3, and DG in the three groups. (a–d) Quantitative analysis. Cortex area, magnification 400×; CA1 area, magnification 400×, scale bar = 25 μm. CA3 and DG areas, magnification 200×, scale bar = 50 μm. The values are mean ± SD. n = 3 animals per group. Cortex: ∗∗ < 0.01, CCH group vs. sham group; && < 0.01, CCH + NBP group vs. sham group. CA1, CA3, DG: No statistically significant differences among the three groups. CCH: CCH 8w; CCH + NBP: CCH 8w + NBP. [file Image_2.TIF]

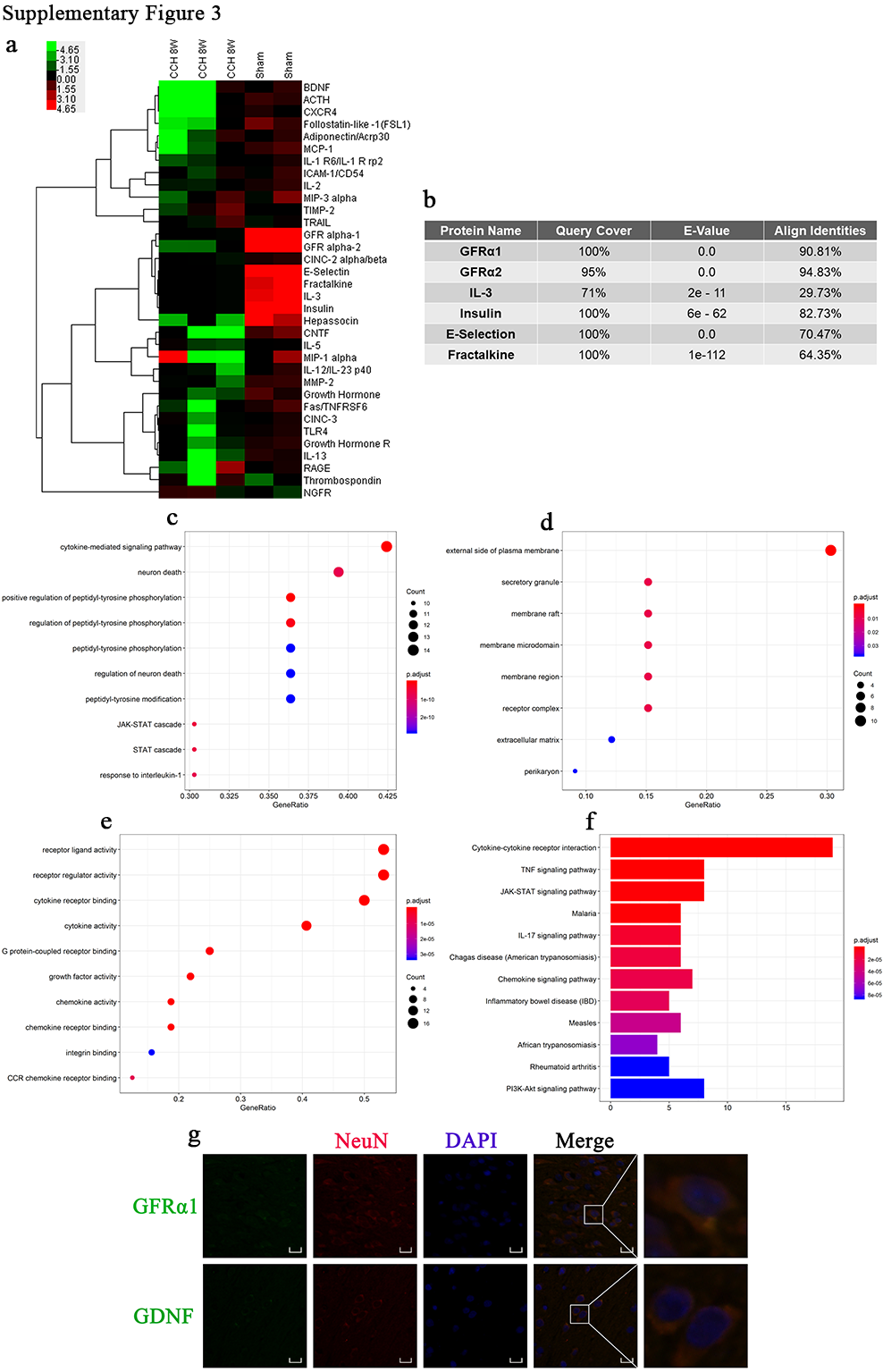

Supplement: FIGURE S3 — Differentially expressed proteins between CCH 8w and sham groups, and biological function analysis. (a) Clustering analysis showed six DEPs (GFRα1, GFRα2, E-Selection, Fractalkine, and I-3, Insulin) were differentially expressed in the hippocampus between CCH 8w and sham groups (p < 0.01). (b) Conservative analysis of six DEPs. The conservation of the six DEPs between Homo sapiens and Rattus norvegicus was analyzed. The results showed that GFRα1 was the most conserved DEP (Query cover = 100%; E-value = 0.0; Align identities score = 90.81%). (c) GO enrichment of DEPs: BP. (d) GO enrichment of DEPs: CC. (e) GO enrichment of DEPs: MF. (f) KEGG enrichment analysis of DEPs. (g) Localization of GFRα 1 and GDNF mRNA as shown by in situ hybridization upon NBP treatment. magnification 400×, scale bar = 25 μm. [file Image_3.TIF]

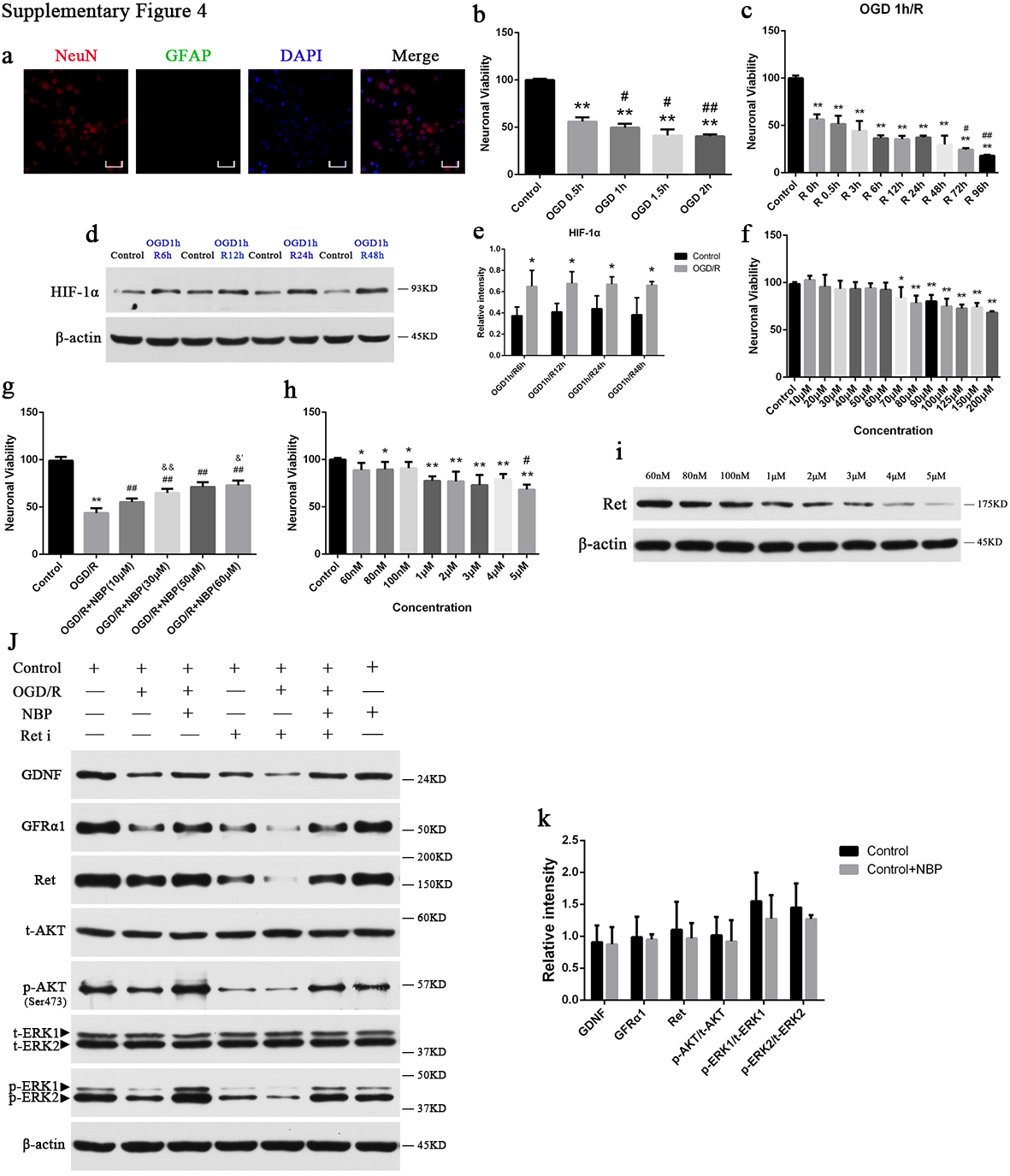

Supplement: FIGURE S4 — (a) Purity test of primary hippocampal neurons. 400× magnification. (b,c) Changes in neuronal viability after OGD/R Neuron viability was measured in primary hippocampal neuron cultures under the following conditions: normal, OGD 0.5 h, OGD 1 h, OGD 1.5 h, OGD 2 h, as well as OGD 1 h/R 0 h, 0.5 h, 3 h, 6 h, 12 h, 24 h, 48 h, 72 h, and 96 h using CCK-8. After OGD or OGD 1 h/R, the viability of neurons had decreased to different degrees (b), ∗∗P < 0.01 compared to sham, #P < 0.05, ##P < 0.01 compared to OGD 0.5 h; (c), ∗∗P < 0.01 compared to sham, #P < 0.05, ##P < 0.01 compared to OGD 1 h/R 48 h. (d,e) Expression of HIF-1α after OGD/R in primary hippocampal neurons. Western blotting results showed that after OGD 1 h/R 6 h, OGD 1 h/R 12 h, OGD 1 h/R 24 h, OGD 1 h/R 48 h, HIF-1α expression was significantly increased. n = 3 per experiments. *P < 0.05, compared to control. (f–i) Experimental screening of drug and reagent concentrations In order to select the optimal concentration of NBP to treat neurons, we screened 13 different drug concentrations, specifically, 10, 20, 30, 40, 50, 60, 70, 80, 90, 100, 125, 150, and 200 μM for 24 h. The results showed that when the drug concentration was below 60 μM, there were no damaging effects on neuronal survival (f). *P < 0.05, ∗∗P < 0.01 compared with normal control. The effect of various concentrations of NBP (10, 30, 50, and 60 μM) on OGD/R (OGD 1 h/R 48 h)-induced neuronal cell damage (g). ∗∗P < 0.01 compared with normal control, ##P < 0.01 compared with OGD/R, &&P < 0.01 compared with 10 μM, &′P < 0.05 compared with 30 μM. In order to select the optimal concentration of Ret inhibitor, we set up 8 concentrations, 60, 80, 100, 1, 2, 3, 4, and 5 μM for 6 h. The results showed that treatment with 60 nM Ret inhibitor for 6 h had a significant adverse effect on neuronal survival. At a concentration of 5 μM for 6 h, the expression of Ret could be significantly inhibited (h). *P < 0.05, ∗∗P < 0.01 compared to control, #P < 0.05 compar [file Image_4.TIF]

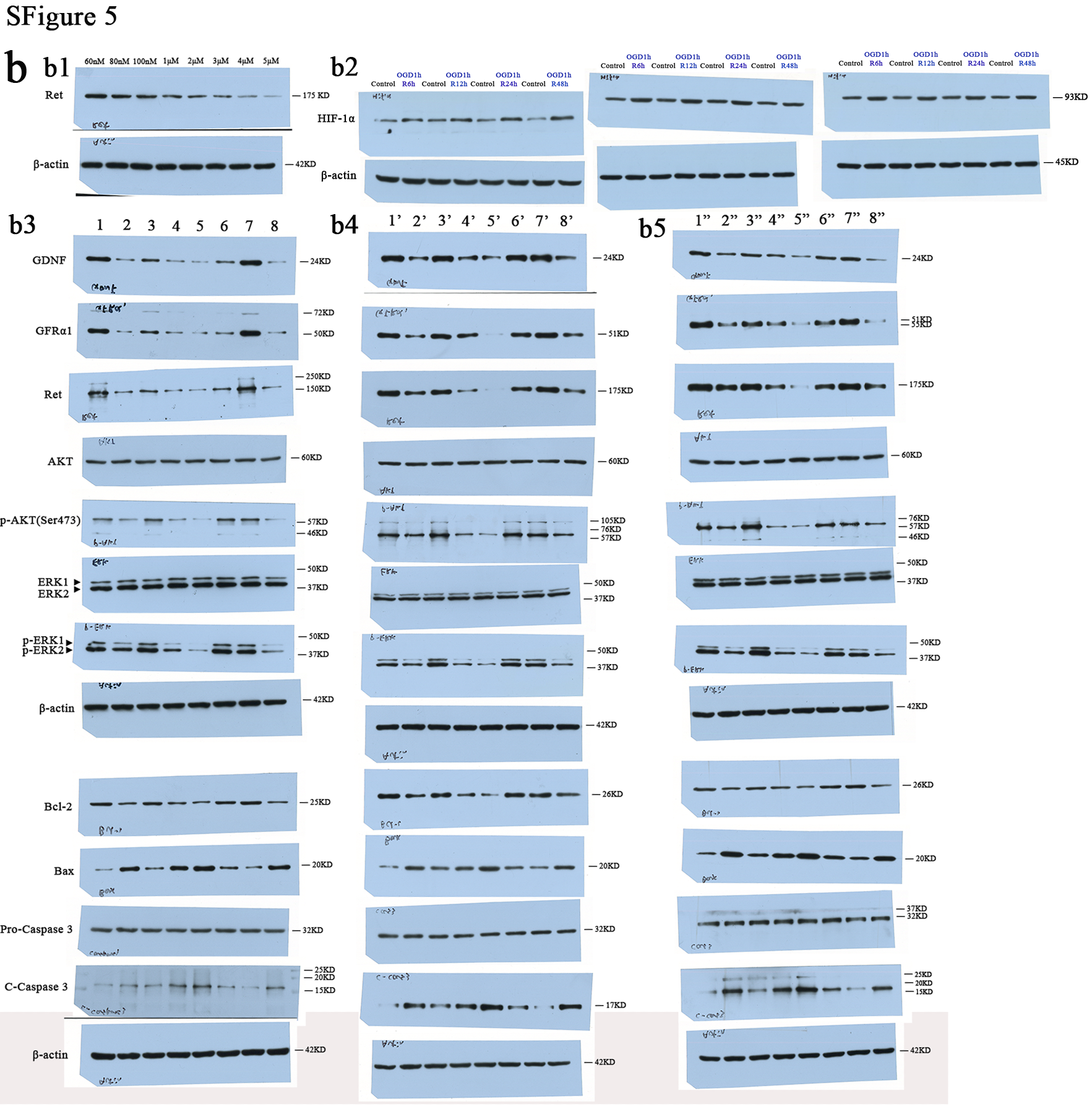

Supplement: Supplementary file 5 [file Image_6.TIF]

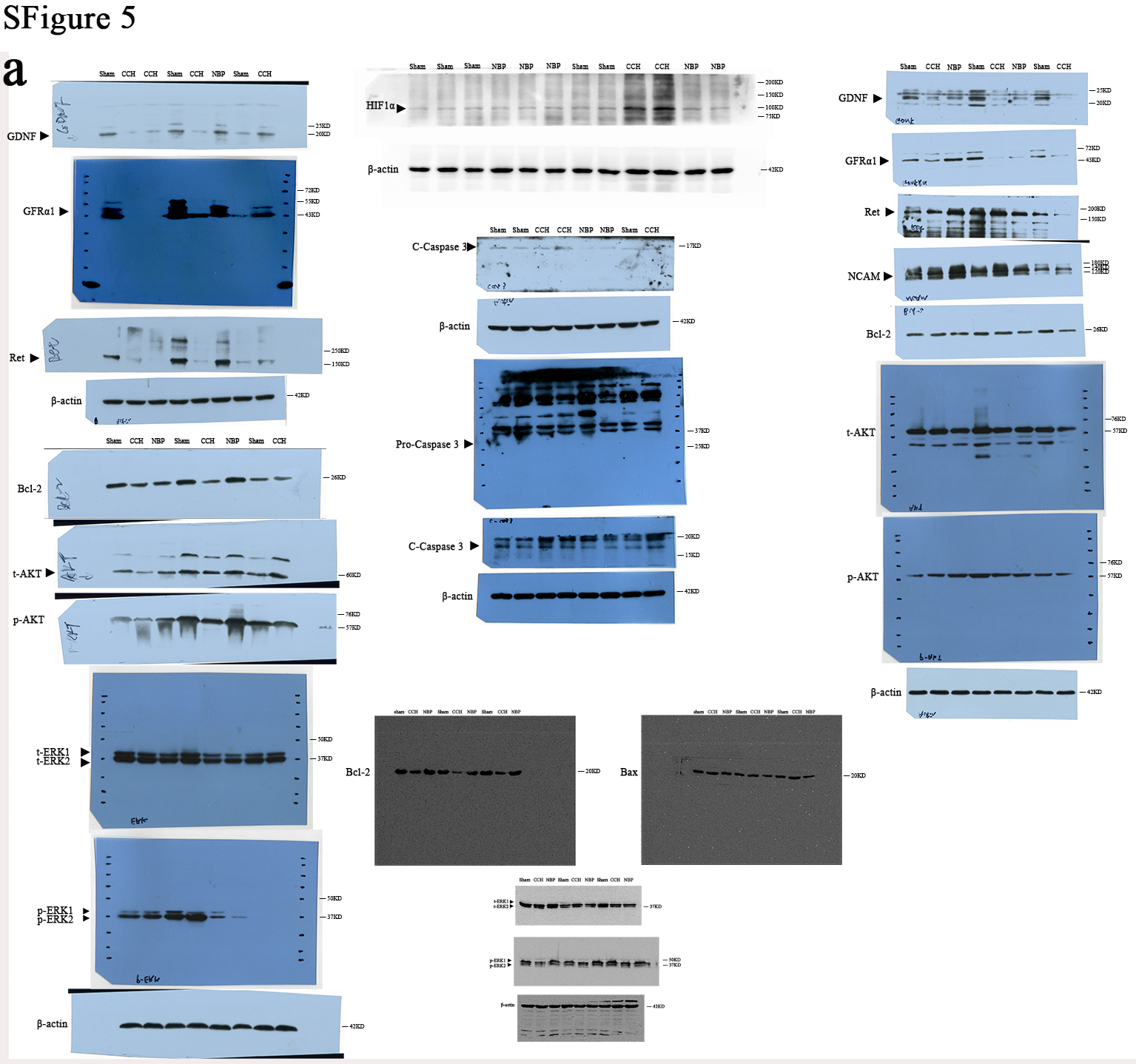

Supplement: FIGURE S5 — (a) Full western blots from in vivo experiments. Proteins were extracted with RIPA from sham, CCH, and NBP groups. Blots were probed with the indicated antibodies. The levels of the protein of interest were normalized to β-actin for every sample. These indicators included GDNF, GFRα1, Ret, NCAM, HIF-1α, pro-caspase 3, cleaved caspase 3, Bcl-2, Bax, t-AKT, p-AKT (Ser473), t-ERK1 (pT202/pY204)/ERK2 (pT185/pY187), p-ERK1 (pT202/pY204)/ERK2 (pT185/pY187). CCH: CCH 8w; NBP: CCH 8w + NBP. (b) Full western blots from in vitro experiments. Proteins were extracted with RIPA from seven groups, including the control, OGD/R, OGD/R + NBP, Ret i, OGD/R + Ret i, OGD/R + Ret i + NBP, and control + NBP. Blots were probed with the indicated antibodies. The levels of the protein of interest were normalized to β-actin for every sample. These indicators included GDNF, GFRα1, Ret, HIF-1α, pro-caspase 3, cleaved caspase 3, Bcl-2, Bax, t-AKT, p-AKT (Ser473), t-ERK1 (pT202/pY204)/ERK2 (pT185/pY187), p- ERK1 (pT202/pY204)/ERK2 (pT185/pY187). (b1) Ret. (b2) HIF-1α. (b3) First trial. (1 control, 2 OGD/R, 3 OGD/R + NBP, 4 Ret i, 5 OGD/R + Ret i, 6 OGD/R + Ret i + NBP, 7 control + NBP, 8 OGD/R.) (b4) Second trial. (1′ control, 2′ OGD/R, 3′ OGD/R + NBP, 4′ Ret i, 5′ OGD/R + Ret i, 6′ OGD/R + Ret i + NBP, 7′ control + NBP, 8′ OGD/R.) (b5) Third trial. (1″ control, 2″ OGD/R, 3″ OGD/R + NBP, 4″ Ret i, 5″ OGD/R + Ret i, 6″ OGD/R + Ret i + NBP, 7″ control + NBP, 8″ OGD/R). [file Image_5.TIF]
