# Supplementary material for: Dl-3-n-Butylphthalide Reduces Cognitive Impairment Induced by Chronic Cerebral Hypoperfusion Through GDNF/GFRα1/Ret Signaling Preventing Hippocampal Neuron Apoptosis
Source: Front Cell Neurosci. 2019 Aug 13;13:351. doi: 10.3389/fncel.2019.00351 (PMC6701226; doi:10.3389/fncel.2019.00351)
Supplement: TABLE S1 — All data from the antibody microarray analyses. [file Table_1.docx]

| **Protein ID** | **EntrezID** | **Sham** | **Sham** | **CCH 8w** | **CCH 8w** | **CCH 8w** | **CCH 8w+NBP** | **CCH 8w+NBP** | **CCH 8w+NBP** |
| --- | --- | --- | --- | --- | --- | --- | --- | --- | --- |
| **Activin A** | 29200 | 0.998099 | 197.2288 | 1.085305 | 1.087878 | 90.51006 | 189.75 | 1.06843 | 1.044074 |
| **ACTH** | 24664 | 243.5361 | 195.4439 | 1.085305 | 1.087878 | 119.823 | 120.5 | 69.98215 | 1.044074 |
| **ADFP** | 298199 | 282.4619 | 305.66 | 90.35168 | 222.471 | 303.4144 | 292.25 | 2.671074 | 1.044074 |
| **Adiponectin/Acrp30** | 246253 | 165.1853 | 284.9108 | 5.426527 | 63.91282 | 293.6434 | 128.5 | 53.9557 | 1.044074 |
| **AMPK alpha 1** | 65248 | 10.97909 | 66.93285 | 55.89323 | 1.087878 | 170.7349 | 91.5 | 44.07273 | 61.86141 |
| **B7-1/CD80** | 25408 | 0.998099 | 43.72946 | 22.79141 | 1.087878 | 64.28271 | 66.5 | 37.12794 | 78.30558 |
| **BDNF** | 24225 | 113.2842 | 206.1532 | 1.085305 | 1.087878 | 173.3062 | 127.5 | 1.335537 | 1.044074 |
| **beta-Catenin** | 84353 | 34.43441 | 25.43448 | 51.00935 | 1.087878 | 207.7617 | 31 | 69.98215 | 8.352595 |
| **basic-FGF** | 54250 | 0.998099 | 0.892438 | 1.085305 | 1.087878 | 1.028523 | 1 | 10.95141 | 1.044074 |
| **beta-NGF** | 310738 | 96.81557 | 48.63787 | 1.085305 | 1.087878 | 157.8783 | 157.25 | 5.876364 | 52.72575 |
| **CCR4** | 171054 | 0.998099 | 0.892438 | 1.085305 | 1.087878 | 1.028523 | 48 | 1.06843 | 1.044074 |
| **CD106** | 25361 | 3226.354 | 3258.737 | 3032.886 | 2004.687 | 3994.271 | 3325.75 | 3990.051 | 3116.04 |
| **CINC-2 alpha/beta** | 171551 | 626.806 | 738.4924 | 448.7738 | 439.2307 | 368.9828 | 1264.75 | 383.8334 | 504.5489 |
| **CINC-3** | 114105 | 417.2053 | 566.6981 | 456.3709 | 57.92949 | 395.9815 | 1059 | 178.1607 | 193.4148 |
| **CNTF** | 25707 | 68.86881 | 133.8657 | 33.10182 | 1.087878 | 1.028523 | 173 | 1.06843 | 1.044074 |
| **CNTF R alpha** | 313173 | 0.998099 | 62.91688 | 30.9312 | 1.087878 | 143.479 | 297.25 | 1.06843 | 1.044074 |
| **CSK** | 315707 | 0.998099 | 39.9366 | 1.085305 | 1.087878 | 1.028523 | 1 | 1.06843 | 1.044074 |
| **CXCR4** | 60628 | 155.9529 | 105.977 | 1.085305 | 1.087878 | 109.2806 | 1 | 1.06843 | 1.044074 |
| **EGFR** | 24329 | 248.7761 | 352.0668 | 337.53 | 1.087878 | 368.2114 | 437.25 | 2.671074 | 117.1973 |
| **EG-VEGF/PK1** | 192205 | 1843.987 | 1788.446 | 2874.974 | 1711.232 | 3050.6 | 1769.75 | 1435.97 | 1213.997 |
| **E-Selectin** | 25544 | 28.44581 | 203.699 | 1.085305 | 1.087878 | 1.028523 | 4 | 1.06843 | 1.044074 |
| **FADD** | 266610 | 0.998099 | 12.94035 | 1.085305 | 1.087878 | 29.05579 | 132.5 | 1.06843 | 60.55631 |
| **Fas Ligand/TNFSF6** | 25385 | 60.88402 | 118.6943 | 1.085305 | 1.087878 | 67.88254 | 157.5 | 16.56066 | 4.176297 |
| **Fas/TNFRSF6** | 246097 | 122.0176 | 219.986 | 49.3814 | 1.631817 | 87.93875 | 107.25 | 1.06843 | 106.4956 |
| **FGF-BP** | 64535 | 119.7718 | 165.9935 | 34.18712 | 119.1226 | 245.8171 | 178.5 | 172.5514 | 8.874632 |
| **Follostatin-like -1(FSL1)** | 79210 | 295.9363 | 137.4355 | 3.798569 | 5.439389 | 73.02516 | 247.5 | 48.61356 | 4.176297 |
| **Fractalkine** | 89808 | 16.96768 | 236.4961 | 1.085305 | 1.087878 | 1.028523 | 48.5 | 33.92265 | 7.830558 |
| **GFR alpha-1** | 25454 | 160.4444 | 82.55051 | 1.085305 | 1.087878 | 1.028523 | 43 | 6.677686 | 12.52889 |
| **GFR alpha-2** | 25136 | 401.7347 | 336.8953 | 1.085305 | 1.087878 | 4.114094 | 568.5 | 8.280331 | 37.06464 |
| **GM-CSF** | 116630 | 994.3558 | 773.7437 | 810.9945 | 488.7291 | 594.4865 | 713 | 432.7141 | 762.1743 |
| **Growth Hormone** | 24391 | 259.0066 | 130.2959 | 97.13483 | 24.20528 | 44.74077 | 543 | 1.06843 | 69.69196 |
| **Growth Hormone R** | 25235 | 125.7604 | 150.822 | 91.70831 | 12.78256 | 61.7114 | 455.5 | 23.23835 | 1.044074 |
| **Hepassocin** | 246186 | 298.4315 | 95.71397 | 1.085305 | 10.06287 | 1.028523 | 1 | 1.06843 | 96.57688 |
| **ICAM-1/CD54** | 25464 | 344.3441 | 708.1495 | 359.2361 | 161.8218 | 483.9203 | 542 | 90.28232 | 427.2874 |
| **ICK** | 84411 | 742.0864 | 525.4229 | 718.2009 | 485.7375 | 690.9106 | 699.75 | 337.3567 | 196.808 |
| **IDE (Insulin Degrading Enzyme)** | 25700 | 697.1719 | 1002.654 | 855.7633 | 503.6874 | 983.7826 | 899 | 428.4403 | 706.8383 |
| **IFN-gamma** | 25712 | 9.481938 | 36.58996 | 1.085305 | 60.37722 | 1.028523 | 48 | 5.609256 | 217.9505 |
| **IL-1 alpha** | 24493 | 41.92015 | 8.92438 | 1.085305 | 1.087878 | 155.8213 | 20 | 426.3035 | 39.93584 |
| **IL-1 beta** | 24494 | 794.9856 | 769.7278 | 597.4606 | 529.7965 | 897.3867 | 772.5 | 480.2592 | 526.7355 |
| **IL-1 R6/IL-1 R rp2** | 171106 | 344.8431 | 493.072 | 116.6703 | 200.7135 | 343.2697 | 411 | 253.485 | 103.3634 |
| **IL-2** | 116562 | 439.912 | 618.4595 | 258.8453 | 234.4377 | 340.4412 | 676.5 | 17.62909 | 128.6822 |
| **IL-3** | 24495 | 20.46102 | 257.4684 | 1.085305 | 1.087878 | 1.028523 | 48 | 1.06843 | 1.044074 |
| **IL-4** | 287287 | 834.161 | 879.0514 | 925.7655 | 436.239 | 665.9689 | 709 | 625.0314 | 727.1978 |
| **IL-5** | 24497 | 1231.155 | 1277.525 | 1372.097 | 599.1487 | 511.4333 | 428.5 | 493.8817 | 506.3761 |
| **IL-6** | 24498 | 126.509 | 44.6219 | 1.085305 | 1.087878 | 1.028523 | 180.5 | 9.615868 | 9.918706 |
| **IL-10** | 25325 | 1192.229 | 1030.989 | 1095.616 | 744.3804 | 705.3099 | 1020.25 | 614.08 | 948.8026 |
| **IL-12/IL-23 p40** | 84405 | 623.8117 | 1074.049 | 626.2212 | 517.5579 | 53.48322 | 345 | 246.5402 | 191.5876 |
| **IL-13** | 116553 | 381.7728 | 310.3453 | 239.5812 | 8.431053 | 90.51006 | 216 | 161.0658 | 104.4074 |
| **Integrin alpha M beta 2** | NA | 6610.907 | 5418.437 | 5367.649 | 5207.671 | 4798.833 | 4866.75 | 4614.548 | 3747.183 |
| **Insulin** | NA | 54.39638 | 173.5792 | 1.085305 | 1.087878 | 1.028523 | 110.5 | 1.06843 | 1.044074 |
| **IP-10** | 245920 | 428.4339 | 800.5169 | 531.257 | 576.5753 | 673.1686 | 676.25 | 522.7293 | 510.5524 |
| **Leptin (OB)** | 25608 | 1.497148 | 0.892438 | 1.085305 | 1.087878 | 81.76761 | 107 | 1.06843 | 104.9295 |
| **LIX** | 60665 | 326.3783 | 251.4444 | 167.6797 | 1.087878 | 418.3519 | 481.25 | 94.28893 | 145.1263 |
| **L-Selectin/CD62L** | 29259 | 59.6364 | 11.15547 | 1.085305 | 88.1181 | 16.97064 | 1 | 1.06843 | 1.044074 |
| **MDC** | 117551 | 1085.931 | 940.4065 | 1084.22 | 766.6819 | 1056.294 | 1397 | 772.7418 | 805.5034 |
| **MIF** | 81683 | 597.1125 | 1056.87 | 1008.791 | 584.1904 | 787.5918 | 997.5 | 677.9187 | 648.8922 |
| **MCP-1** | 24770 | 281.9629 | 406.7286 | 1.085305 | 51.40223 | 156.8498 | 82.5 | 91.35075 | 385.2634 |
| **MIP-1 alpha** | 25542 | 25.95057 | 179.38 | 621.066 | 1.087878 | 1.028523 | 30.75 | 117.5273 | 125.0279 |
| **MIP-2** | 114105 | 9.980987 | 112.8934 | 174.1915 | 1.087878 | 1.028523 | 50.25 | 51.01752 | 1.044074 |
| **MIP-3 alpha** | 29538 | 71.86311 | 380.1786 | 22.79141 | 80.23099 | 204.1619 | 194.5 | 108.7127 | 3.65426 |
| **MMP-2** | 81686 | 249.5247 | 271.5243 | 132.4073 | 139.7923 | 33.42701 | 159.75 | 40.06612 | 1.044074 |
| **MMP-8** | 63849 | 0.998099 | 0.892438 | 1.085305 | 1.087878 | 1.028523 | 67.5 | 1.06843 | 1.044074 |
| **MMP-13** | 171052 | 0.998099 | 20.74918 | 1.085305 | 1.087878 | 1.028523 | 1 | 1.06843 | 1.044074 |
| **MuSK** | 81725 | 125.2614 | 49.75342 | 1.085305 | 1.087878 | 1.028523 | 17.5 | 1.06843 | 78.56659 |
| **Neuropilin-2** | 81527 | 1437.013 | 1090.113 | 1190.309 | 966.8514 | 1233.2 | 1801.25 | 786.8985 | 1352.076 |
| **NGFR** | 24596 | 260.0047 | 138.7741 | 461.2548 | 500.1518 | 189.7626 | 300 | 300.763 | 382.1312 |
| **Orexin A** | 25723 | 752.0674 | 822.1585 | 576.8398 | 596.1571 | 1050.38 | 489.5 | 698.7531 | 1033.895 |
| **Osteopontin/SPP1** | 25353 | 36.92965 | 0.892438 | 1.085305 | 1.087878 | 1.028523 | 1 | 1.06843 | 1.044074 |
| **PDGF-AA** | 25266 | 346.8393 | 393.1189 | 184.5019 | 135.1688 | 487.7772 | 197.5 | 196.324 | 414.2365 |
| **Prolactin R** | 24684 | 237.0484 | 259.4763 | 25.50468 | 228.4543 | 455.3787 | 203 | 163.4698 | 121.1126 |
| **RAGE** | 81722 | 48.90684 | 64.92486 | 14.10897 | 1.087878 | 335.2986 | 49.75 | 51.81884 | 1.044074 |
| **RALT/MIG-6** | 313729 | 96.56605 | 530.1082 | 427.339 | 160.19 | 166.1065 | 306.75 | 47.27802 | 359.4226 |
| **RELM gamma (RELM gamma)** | 288135 | 0.998099 | 122.9333 | 1.085305 | 1.087878 | 1.028523 | 68.5 | 45.94248 | 28.19001 |
| **Resistin** | 246250 | 40.92205 | 112.8934 | 1.085305 | 1.087878 | 1.028523 | 107.25 | 1.06843 | 1.044074 |
| **TAL1A** | 252878 | 0.998099 | 55.77737 | 1.085305 | 1.087878 | 1.028523 | 1 | 1.06843 | 115.1092 |
| **TGF-beta1** | 59086 | 109.2918 | 89.69002 | 1.085305 | 50.58632 | 1.028523 | 40 | 1.06843 | 62.64446 |
| **TGF-beta2** | 81809 | 0.998099 | 10.26304 | 1.085305 | 1.087878 | 1.028523 | 1 | 1.06843 | 1.044074 |
| **TGF-beta3** | 25717 | 0.998099 | 0.892438 | 1.085305 | 1.087878 | 1.028523 | 253.25 | 24.03967 | 1.044074 |
| **Thrombospondin** | 445442 | 83.84029 | 309.0067 | 371.1745 | 1.087878 | 567.2307 | 359.75 | 216.0899 | 1.044074 |
| **TIE-2** | 89804 | 328.8735 | 458.0438 | 108.5305 | 674.2123 | 375.411 | 400 | 203.803 | 470.0945 |
| **TIMP-1** | 116510 | 0.998099 | 37.03618 | 8.139791 | 2.447725 | 1.028523 | 58 | 52.62017 | 86.65817 |
| **TIMP-2** | 29543 | 193.1321 | 194.5515 | 88.45239 | 246.9483 | 707.367 | 505.75 | 51.28463 | 83.78697 |
| **TIMP-3** | 25358 | 167.4311 | 203.0296 | 1.085305 | 47.05072 | 393.4102 | 263.5 | 3.205289 | 1.044074 |
| **TLR4** | 29260 | 249.7742 | 314.5844 | 220.5883 | 2.991664 | 203.3905 | 196.5 | 227.5755 | 1.044074 |
| **TNF-alpha** | 24835 | 742.5854 | 806.9871 | 567.8861 | 335.0664 | 825.39 | 775.75 | 423.0982 | 420.762 |
| **TRAIL** | 246775 | 1598.705 | 2143.636 | 1955.72 | 1589.661 | 5042.079 | 3005.5 | 1320.045 | 1252.106 |
| **TROY** | 290300 | 2565.114 | 2390.618 | 3856.633 | 3430.623 | 2519.882 | 2694 | 2416.521 | 3013.721 |
| **Ubiquitin** | 192255 | 1792.835 | 2079.604 | 1632.842 | 1612.235 | 1131.633 | 1919 | 1321.113 | 1501.901 |
| **VEGF** | 83785 | 1378.125 | 2101.915 | 1705.557 | 1111.811 | 1323.967 | 709.5 | 1221.749 | 1002.311 |
| **VEGF-C** | 114111 | 0.998099 | 0.892438 | 1.085305 | 1.087878 | 1.028523 | 11 | 1.06843 | 8.874632 |
